# The Malawi Developmental Assessment Tool (MDAT): The Creation, Validation, and Reliability of a Tool to Assess Child Development in Rural African Settings

**DOI:** 10.1371/journal.pmed.1000273

**Published:** 2010-05-25

**Authors:** Melissa Gladstone, Gillian A. Lancaster, Eric Umar, Maggie Nyirenda, Edith Kayira, Nynke R. van den Broek, Rosalind L. Smyth

**Affiliations:** 1 Department of Paediatrics, College of Medicine, Blantyre, Malawi; 2 Postgraduate Statistics Centre, Department of Mathematics and Statistics, Lancaster University, Lancaster, United Kingdom; 3 Department of Community Health, College of Medicine, Blantyre, Malawi; 4 Wellcome Trust Research Labs, Blantyre, Malawi; 5 Liverpool School of Tropical Medicine, Liverpool, United Kingdom; 6 School of Reproductive and Developmental Medicine, Institute of Child Health, University of Liverpool, Liverpool, United Kingdom; University College London, United Kingdom

## Abstract

**Background:**

Although 80% of children with disabilities live in developing countries, there are few culturally appropriate developmental assessment tools available for these settings. Often tools from the West provide misleading findings in different cultural settings, where some items are unfamiliar and reference values are different from those of Western populations.

**Methods and Findings:**

Following preliminary and qualitative studies, we produced a draft developmental assessment tool with 162 items in four domains of development. After face and content validity testing and piloting, we expanded the draft tool to 185 items. We then assessed 1,426 normal rural children aged 0–6 y from rural Malawi and derived age-standardized norms for all items. We examined performance of items using logistic regression and reliability using kappa statistics. We then considered all items at a consensus meeting and removed those performing badly and those that were unnecessary or difficult to administer, leaving 136 items in the final Malawi Developmental Assessment Tool (MDAT). We validated the tool by comparing age-matched normal children with those with malnutrition (120) and neurodisabilities (80). Reliability was good for items remaining with 94%–100% of items scoring kappas >0.4 for interobserver immediate, delayed, and intra-observer testing. We demonstrated significant differences in overall mean scores (and individual domain scores) for children with neurodisabilities (35 versus 99 [*p*<0.001]) when compared to normal children. Using a pass/fail technique similar to the Denver II, 3% of children with neurodisabilities passed in comparison to 82% of normal children, demonstrating good sensitivity (97%) and specificity (82%). Overall mean scores of children with malnutrition (weight for height <80%) were also significantly different from scores of normal controls (62.5 versus 77.4 [*p*<0.001]); scores in the separate domains, excluding social development, also differed between malnourished children and controls. In terms of pass/fail, 28% of malnourished children versus 94% of controls passed the test overall.

**Conclusions:**

A culturally relevant developmental assessment tool, the MDAT, has been created for use in African settings and shows good reliability, validity, and sensitivity for identification of children with neurodisabilities.

*Please see later in the article for the Editors' Summary*

## Abstract 2

Melissa Gladstone and colleagues evaluate the reliability and validity of an assessment tool for evaluating child development in rural African settings.

## Editors' Summary

**Background:**

Babies can do very little when they are first born. But, gradually, over the first few years of life, they learn to walk and run (gross motor skills), they learn to manipulate objects with their hands (fine motor skills), they learn to communicate with words and gestures (language skills), and they learn how to interact with other people (social skills). For each of these skill “domains,” experts have identified “developmental milestones,” skills that are acquired by a specific age if development is proceeding normally. So, for example, by one year, a child should be able crawl, put objects into a container, respond to simple verbal requests, and enjoy imitating people during play. Every child attains the various developmental milestones at a slightly different age but their overall development can be monitored using a series of simple tests (items) covering each of the developmental domains. Clinicians and teachers can use these “developmental assessment tools” to identify children whose development deviates significantly from the norm. It is important to identify these children as young as possible because early intervention can help them reach their full developmental potential.

**Why Was This Study Done?:**

In developing countries, poverty, poor health, and malnutrition are responsible for millions of children failing to reach their developmental potential. But because developmental assessment tools have mainly been designed and validated in western, developed countries, they contain many items that are alien to children in non-western cultures (for example, the use of knives and forks for eating and the use of specific gestures). They cannot, therefore, accurately assess whether a child living in, for example, a rural area of Africa, is developing normally. In this study, the researchers describe the creation and testing of a culturally appropriate developmental assessment tool for use in rural Africa—the Malawi Developmental Assessment Tool (MDAT)—from a 162-item draft tool (MDAT Draft I) that they previously developed from Denver II, an assessment tool widely used in developed countries.

**What Did the Researchers Do and Find?:**

The researchers assessed the “face validity” (do the items look acceptable to untrained judges?) and “content validity” (does the tool examine all the domains it is meant to measure?) of MDAT Draft I and modified it to produce MDAT Draft II. After piloting this version on 80 children in rural Malawi, they modified it further to produce MDAT Draft III, which was used to assess 1,426 normal children aged 0–6 years from rural Malawi and to derive age-standardized norms for each item. After statistically analyzing the performance of each item in MDAT Draft III, all the items were considered at a consensus meeting, and items that were badly performing, unnecessary, and difficult to administer were removed, leaving 136 items (MDAT). The researchers then validated MDAT by using it to assess children with neurodisabilities (disorders of the nervous system that impair normal functioning) and children with delayed development because of malnutrition. The tool was reliable (different testers got similar results for individual children and individual testers got similar results when they retested specific children), sensitive (it correctly identified most children with a neurodisability or delayed development), and specific (it correctly identified most children who were developing normally; that is, it did not give false-positive results).

**What Do These Findings Mean?:**

These findings show that MDAT is a culturally relevant assessment tool that reliably identifies children with neurodisabilities and delayed development in rural Malawi. Importantly, they also provide a detailed illustration of how to create and validate a culturally relevant assessment tool. Although MDAT is likely to be applicable in other similar settings, further research is needed to test its generalizability and to test whether it will work in children with more subtle developmental problems. MDAT, the researchers note, should be useful as a clinical tool for the early identification of neurodisabilities and as an outcome measure in clinical trials of interventions designed to improve child development. However, they stress, because developing countries have limited resources available for screening and for helping children whose development is delayed or disrupted, for now tools like MDAT are more likely to be used for research studies than for routine developmental assessments in Malawi and other African countries.

**Additional Information:**

Please access these Web sites via the online version of this summary at http://dx.doi.org/10.1371/journal.pmed.1000273.

- The World Health Organization has information on disability, prevention, and management in children and adults worldwide
- UNICEF has a site on early childhood and in particular, provides information on programming experiences for early child intervention programs worldwide
- Disability World is a website for international views and perspectives on disability worldwide. It provides information and links about the worldwide state of disability in children and adults in developing countries
- Source, the International Information Support Centre has a good website of information about disability, inclusion, and development in children with links to many other sources of information
- Wikipedia has a page on child development (note that Wikipedia is a free online encyclopedia that anyone can edit; available in several languages)
- The US Centers for Disease Control and Prevention provides information on developmental screening and on developmental milestones
- The American Academy of Pediatrics also provides information on developmental stages and on developmental milestones
- The UK National Health Service Choices site has an interactive guide to child development
- MedlinePlus has links to further resources on infant and toddler development (in English and Spanish)

## Introduction

Worldwide, poverty, poor health and nutrition are responsible for more than 200 million children under 5 y of age failing to reach their developmental potential [1]. We know that such outcomes could be prevented if early intervention programmes were available for these children [2]. However, the implementation of these internationally funded programmes is critically dependent on tools to assess child development, and there is a dearth of such tools for use in non-Western settings. Programmes and studies using development as an outcome measure in resource-limited countries have tended to use Western assessment tools [3]. Many are simply translated [4] or adapted [5], with limited validation [6] before use. This approach may enable some comparison between groups, but it will not provide robust outcome measures because these tools contain many items alien to children of a non-Western culture [7]. More recently, some tools have been adapted and validated, and normal reference ranges or scores for ages to assess attainment have been developed. These tools have been created for children of a limited age range, [8], have been based solely on urban children [9], or have excluded important domains of development such as language and social skills [10].

The aim of this study was to create a culturally appropriate developmental assessment tool, the Malawi Developmental Assessment Tool (MDAT), for use in rural Africa. In a preliminary study we evaluated the use of Western developmental items in a rural Malawian setting [11]. We discovered that a high proportion of gross motor 33/34 (97%), language 32/35 (91%), and fine motor 27/34 (79%) items were reliable and showed a good fit with logistic regression. The social items 18/35 (51%), however, performed less well and many were judged to be culturally inappropriate. This stimulated us to conduct a qualitative study addressing concepts and ideas of child development with ten focus groups of villagers and two focus groups of professionals in Malawi [12]. While all domains were discussed, gross motor and social milestones were the main domains of interest. Concepts and ideas from this study were then used to generate new items and modify items from the preliminary study. Examples of concepts used were “carrying items on head,” “body healthy and flexible,” “carrying out duties and chores,” “sharing,” and “taking up leadership roles.” All items once created or modified from the preliminary tool were tested in a large community study and normal reference ranges were found for each item. Final items were subsequently selected at a consensus meeting. By these methods we have created the MDAT, a simple to use, reliable, valid, and easily accessible tool for use by community health workers and researchers looking at developmental outcomes of children in sub-Saharan Africa.

## Methods

### Creation of a Culturally Appropriate Developmental Assessment Tool (Pilot Phase)

**Figure 1:**
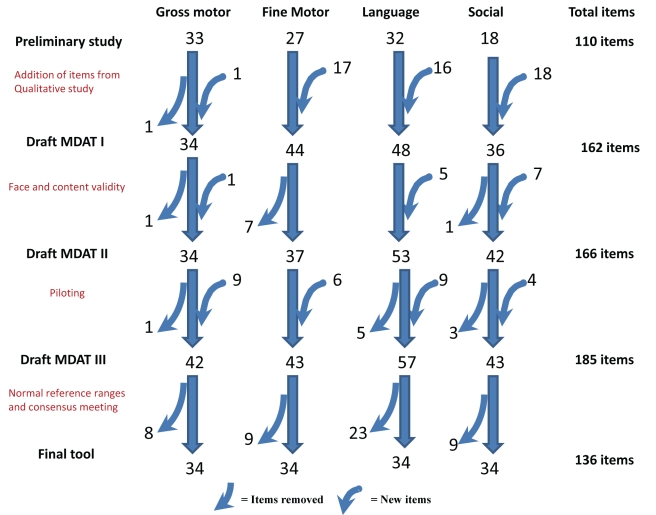
Stages in creation of final MDAT tool. Draft MDAT I created out of 110 items from the preliminary study with the addition of 52 items from the qualitative study, as well as the modification of some items. Draft MDAT II created after face and content validity with addition of 13 items and eight items removed as well as the modification of some items. Draft MDAT III created after piloting where nine gross motor, six fine motor, nine language, and four social items were added or modified, and one gross motor, five language, and three social items were removed. The Final MDAT tool consisted of 136 items with 34 in each domain having had eight gross motor, nine fine motor, 23 language, and nine social items removed.

**Figure 2:**
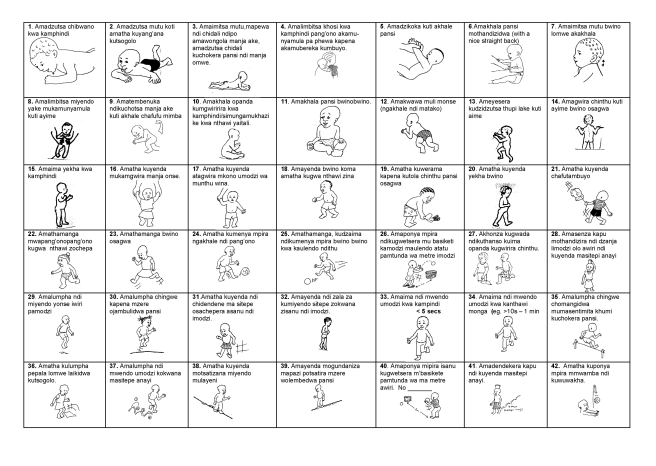
Example of the Draft MDAT III (gross motor domain).

As shown in Figure 1, at the start of this study, MDAT Draft 1 contained 162 items. This draft was created from items in the preliminary study as well as from the qualitative study [11],[12]. We ensured consistency and clarity of items by translating and back translating the tool with the help of a language expert from the University of Malawi. Many items were then illustrated with a picture drawn by a Malawian artist (CZ) (Figure 2). We prepared a small basket of props to be used with the questionnaire (Figure S1). We then assessed face validity (where items were reviewed by untrained judges to see whether they think the items look acceptable) and content validity (the subjective measurement of the comprehensiveness to which an instrument appears logically to examine the characteristics or domains it is intended to measure) [13] through group discussions with six research midwives and ten Malawian medical students. In assessing face validity, individual discussions were also carried out with two of the investigators (EU, MN) and a language expert. These individuals commented on each item and whether the items were understandable and relevant to the Malawian population. At this phase of validation, some items were removed and some added, producing MDAT Draft II (Figure 1).

MDAT Draft II was then piloted on 80 children in two stages over a 6-wk period. Pilot assessments were observed by three investigators (MG, EU, and MN) and there were group discussions every 2 wk with the research midwives. The three investigators met three times during piloting and some items were added to improve clarity or precision and other items were removed either because they were not felt to be discriminatory enough in assessing child development or they were difficult to carry out in the field [14]. At this stage MDAT Draft III was produced with any new items added having face and content validation and being re-piloted. An example of the gross motor domain is shown in Figure 2.

The study protocol complied with the principals of the Helsinki Declaration [15]. The research midwives explained the purpose of the developmental assessment to each child's parent or carer and obtained their informed consent to participation in the study. The study received ethical approval from the College of Medicine Research Ethics Committee in Malawi and the Liverpool School of Tropical Medicine Research Ethics Committee in the UK as well as each of the local health centres where the study took place.

### Assessing the Performance of Items and Establishing Normal Reference Ranges in a Large Sample

**Figure 3:**
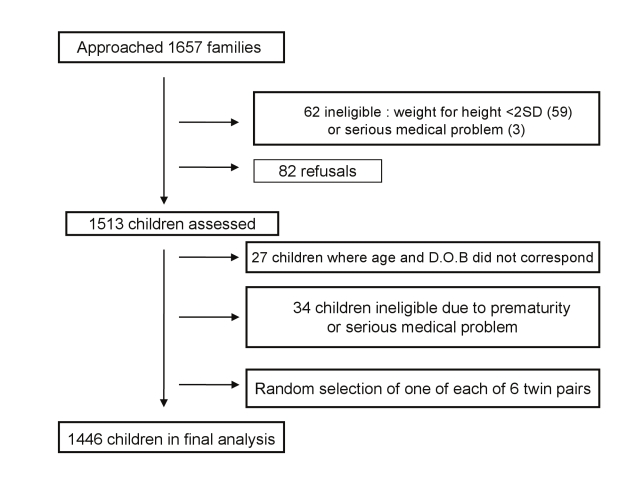
Flow diagram of the recruitment of families and children for the MDAT study.

To test the performance of MDAT Draft III, we recruited and assessed 1,513 children from four sites in the Southern region of Malawi. These were three rural and one semi-urban site (Namitambo, Mikolongwe, Nguludi, and Bangwe), which were all taking part in an antenatal trial with the same research midwife team [16]. Assessments occurred over a 1-y period from June 2006 until July 2007 using the team of six research midwives in local antenatal clinics in each of these areas. Normal healthy children of mothers attending clinic (one per family) between the ages of 0 and 6 y were included. Those with significant malnutrition (weight for height Z score <−2 using WHO criteria [17]), significant medical problems, prematurity of 32 wk or less (reported or measured on antenatal ultrasound), or significant neurodisability were excluded. In all cases, we ensured that they were receiving appropriate medical support. A decision was made to exclude these children from the “normal population” as the aim was to create a developmental assessment tool that identified children with developmental delay. We gathered sociodemographic characteristics using the same questions as the Malawi Demographic Health Survey (MDHS) [18]. We recruited children by asking one in every three mothers in clinic to bring one child to their next appointment. We used a quota sampling technique similar to that used by the Denver II [19] where target numbers of children for 34 age groups were sought (Table S1). Children's ages were determined from available birth data or the “health passport” that mothers in Malawi carry with them for all health appointments. Once we had recruited enough children of a particular age range, no more children of that age range were invited to participate. We then targeted ages where there were inadequate numbers by asking mothers to only bring children of those ages. We approached 1,657 families (Figure 3). 82 families refused and 62 children were ineligible due to serious medical problems as listed above, resulting in 1,513 children in the final assessment. 67 (4.4%) of these were then excluded prior to analysis (Figure 3) leaving 1,446 children in the final analysis. A subsample from this population were recruited for reliability testing.

The assessment using the new tool (MDAT Draft III with 185 items) took approximately 35 min in a quiet location, often outdoors. Five to seven children were assessed in a morning session by two to three research midwives at two of the four different sites each day. Where possible, items were directly observed, but items were accepted on report if the mother was very clear that the child could do the item and there was no doubt when assessing associated areas of development. We scored items as pass or fail, and if the child was uncooperative or unwell, items were scored as “don't know.” Items were assessed until the child failed seven consecutive items [20],[21]. The data for each item were then fitted using logistic regression and normal reference ranges were established (see statistical analysis section).

### Reliability

Children were invited to participate in reliability testing as follows. The first child on the testing day was assessed for interobserver immediate reliability, the second child for interobserver delayed reliability, and the third child for intra-observer delayed reliability. We measured interobserver immediate reliability by assessing the same child independently on the same occasion by two observers (56 children). Interobserver delayed reliability was measured by observing the same child independently on the same day at different times by two observers (52 children). We measured intra-observer delayed reliability by the same observer assessing the same child 2 wk apart (124 children). Reliability testing was carried out on all 185 items in the Draft MDAT III.

### Final Evaluation of Items by Consensus

An expert panel consisting of two Malawian paediatricians, two British paediatricians, and a statistician (MN, Mac Mallewa, MG, RLS, and GAL) reviewed the results and decided which items should remain, which should be further modified, and which removed as previously described [11]. Items were evaluated at these meetings in terms of their fit in a logistic regression, their reliability, subjective ratings, and the effect of gender in the logistic regression. We wanted (as much as possible) items with a good fit, good to excellent reliability (kappa >0.6), few problems when rated subjectively, and no effect of gender. As there were some items where the age ranges for attainment were exactly the same, the consensus meeting used this forum to also choose only one of these items in any one domain. The selection procedure through consensus has been described elsewhere in more detail [11].

### Validity

Once the final set of items was chosen, children were then scored in two ways. Firstly a score was generated by a categorical pass or fail assessment, and each score was used to validate the tool in a series of tests. All items relevant to the age of testing were scored in a similar way to the Denver II screening test [19]. If the child failed two items or more in any one domain at the chronological age at which 90% of the normal reference population would be expected to pass, then they failed the test. Secondly, a continuous score was obtained by adding up the total number of items passed by the child per domain and in total. These scores varied with the age of the child.

Both sets of scores were then used to validate the tool by comparing firstly with a group of children with neurodisability. We recruited 80 children up to 6 y of age with known neurodisabilities from the “Feed the Children” centre for children with disabilities (previously Cheshire Homes) in Blantyre [22]. Exclusions from this group were children unwell at time of examination, those with severe malnutrition (as previously defined), and any blind or deaf children. A second comparison group was 120 children up to 6 y of age with marasmus (height/weight <80% expected), as there is good evidence that these children often have moderate developmental delay [23],[24]. Within this group, children with fevers or other illnesses (including HIV sero-positivity) were excluded. HIV testing was routinely performed in the malnutrition unit. Each of these groups was compared with a subset of age- and sex-matched children from the normal study population. This sample was chosen because of practicality issues and time constraints. To avoid bias, the comparison group was selected randomly (within those of the same sex and age to one decimal place) by a computer-generated random number list.

### Data Entry and Statistical Analysis

All data were double entered by a data entry team with any discrepancies and outlying results reviewed. Data were analysed using Microsoft Access version 7.0 and SPSS for Windows version 12, Stats-direct, STATA version 8 and Epi Info computer programs for the analysis. We measured socioeconomic status in quintiles through principal components analysis of multiple assets following methods from the World Bank [25]–[27]. We determined height and weight for age (HAZ and WAZ) through Epi Info using US Centers for Disease Control reference data [28],[29].

**Figure 4:**
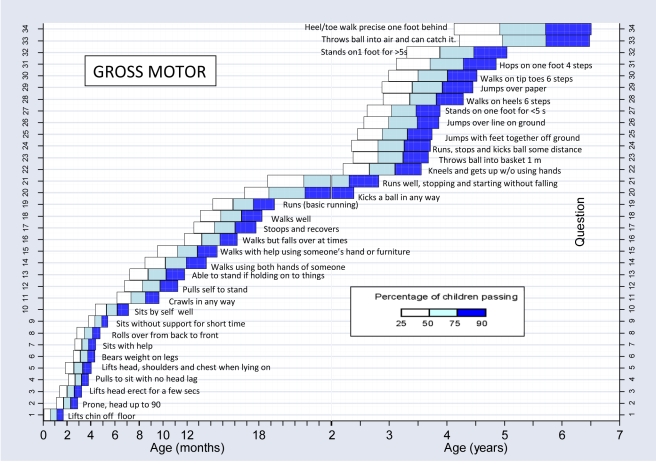
Normal reference values for gross motor milestones.

We constructed normal reference ranges for the children passing items using logistic regression analysis with decimal age as the explanatory variable. A logistic regression analysis is one where a prediction is made about the probability of an event taking place by fitting the data to a logistic curve. In this case, this would be the probability of carrying out a certain item of development e.g. “walks well” at certain decimal ages. The fitted values from the model for each item were plotted against the observed data and graphs were drawn for each item. To determine whether or not the fitted curve was a sufficiently good representation of the data, it was visually assessed for each graph but also statistically assessed. The goodness-of-fit statistic was calculated for each fitted curve and for any item where the fit was significantly poor at the 5% significance level [30], refitting was done using triple split spline regression [31],[32]. To do this, the ages corresponding to the 35th and 65th percentiles were calculated from the original fit to determine the cut points, and three logistic curves were then fitted, one for each region. This calculation is described in more detail in a previous paper [11]. Using the predicted probabilities found from the logistic regression analyses, the ages corresponding to 25%, 50%, 75%, and 90% percent of the children passing were determined for each item. These numbers were then used to plot the age norms of achievement of each milestone in a box-type representation in graphs similar to the procedure described for the Denver II (see Figures 4–7). In a further exploratory analysis, we added other explanatory variables (sex, socioeconomic status, and height for age [HAZ] and weight for age [WAZ] Z scores) to assess their effect on the probability of passing an item.

**Figure 5 pmed-1000273-g005:**
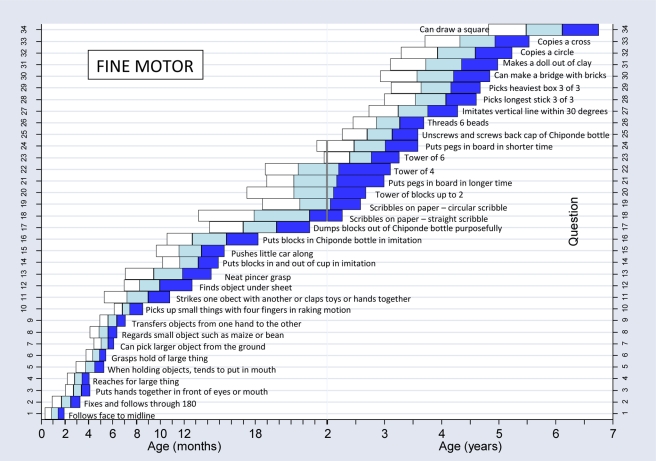
Normal reference values for fine motor milestones.

**Figure 6 pmed-1000273-g006:**
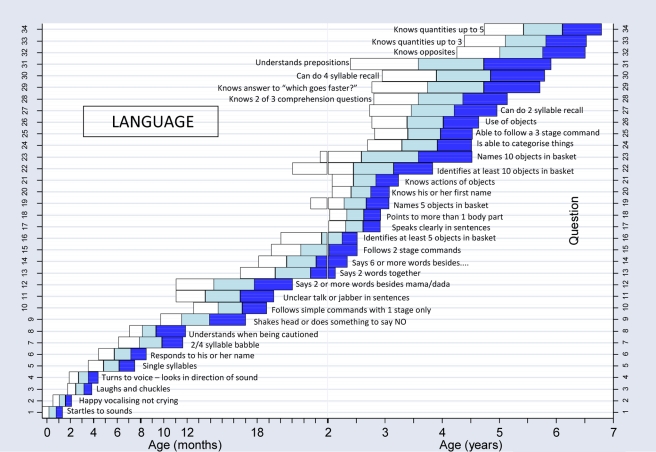
Normal reference values for language milestones.

**Figure 7:**
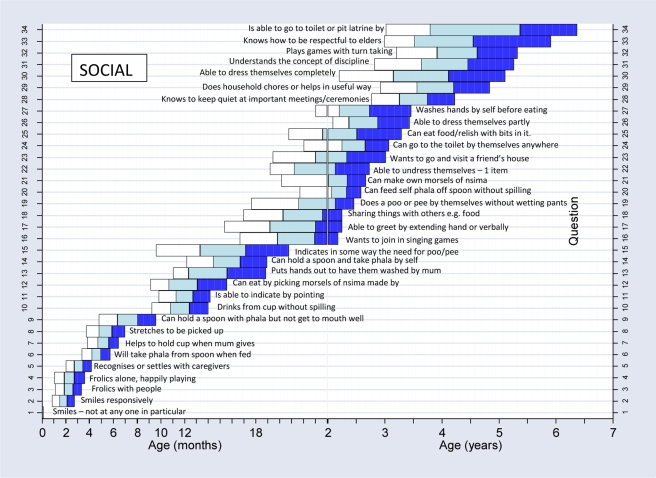
Normal reference values for social milestones.

Reliability was measured using kappa (k) statistics as well as percentage agreement to assess observer agreement for each question. Positive values of 0 to <0.2 indicate poor agreement, >0.2 to 0.4 fair agreement, >0.4 to 0.6 moderate agreement, >0.6 to 0.8 good, and >0.8 to 1 very good agreement [33].

To compare statistically the differences in numbers of pass/fails achieved by the different groups in the construct validity assessment, a paired McNemar's test was used. We used paired *t*-tests to compare the numerical scores. Sensitivity and specificity were calculated for children with neurodisabilities in comparison to normal children, as by definition, children with neurodisabilities clearly should fail a test assessing normal development.

## Results

### Characteristics of Population for MDAT

Demographic data (Table 1) demonstrate the MDAT population was very similar in socioeconomic status to the national average, although the MDAT population had a higher number of mothers with some secondary education (23% versus 10%) and a lower number with no education (11% versus 25%). The MDAT population was nutritionally less stunted than the national average, with a lower proportion of HAZ scores less than 2 or 3 standard deviations (SDs) (<2 SD) below the norm (38% compared to 48%) and for WAZ scores (15% compared to 21%) were <2 or 3 SD below the norm.

**Table 1:** Comparison of socioeconomic data and nutritional status of the MDAT and MDHS 2004 [18].

Characteristic	MDAT Study Population, *n* = 1,426, *n* (%)	MDHS Population, *n* = 8,522, *n* (%)
**Wealth quintile**		
Lowest	305 (21)	1,680 (20)
Second	258 (18)	1,813 (21)
Middle	290 (20)	1,916 (22)
Fourth	306 (21)	1,732 (20)
Highest	284 (20)	1,380 (17)
Not known/missing data	3 (0)	1 (0)
**Nutritional status of children**		
**Height for age**		
Height for age >−2SD (normal range)	858 (59)	4,453 (52)
Height for age below −2SD to −3SD (stunted)	298 (21)	2,177 (26)
Height for age below −3SD (severe stunting)	237 (17)	1,892 (22)
Height for age (total) below −2SD (stunted and severely stunted)	535 (38)	4,069 (48)
Not known/missing data	53 (4)	0
**Weight for age**		
Weight for age >−2SD (normal range)	1,187 (82)	6,647 (78)
Weight for age below −2SD to −3SD (underweight)	185 (13)	1,488 (17)
Weight for age below −3SD (severely underweight)	26 (2)	387 (4)
Weight for age (total) below −2SD (underweight and severely underweight)	211 (15)	1,775 (21)
Not known/missing data	48 (3)	0
**Educational status of mother**		
No education	165 (11)	2,130 (25)
Primary	928 (64)	4,994 (59)
Secondary	331 (23)	841 (10)
Not known/missing data	22 (2)	557 (6)

### Face and Content Validity and Piloting

Initial validation of the Draft MDAT I demonstrated good content and face validity (Figure 1). At this stage, after comments from discussants, 13 items were added to the gross motor, language, and social domains as it was felt there were too few items for certain age ranges. Eight items were also removed in the fine motor and gross motor domains as they were not felt to be culturally appropriate or suitable for testing. The MDAT appeared to assess development in children in ways that were felt to be important. Discussants were happy that the questionnaire examined the various domains of development in a comprehensive and logical fashion and that it was representative and relevant to developmental milestones of children in a Malawian setting.

After face and content validation, the tool was piloted. At this stage, nine language items were added or modified from the previous version for clarity and consistency of items. Nine gross motor items of increasing difficulty were added as it was found that many of the older children were able to do all items in the gross motor section earlier than expected. This was also the case with four social items. Six fine motor items were also added at this stage, often these were items that could be tested differently at different ages and therefore were separated into subsections and consequently different questions, to decrease ambiguity on testing. For example, the item “puts pegs into board” was subdivided as “puts pegs into board in up to 30 secs” and “puts pegs into board in up to 2 minutes.”

### Performance of Items and Normal Population Reference Ranges

Information regarding the final items and how they performed in terms of logistic regression as well as with the additional explanatory variables are shown in Table 2. There were no items in the gross motor domain that had poor goodness of fit in the logistic regression analysis, whereas 50% of items in the social domain needing refitting using splines. A few items (eight) showed gender differences in the analysis but were kept in the tool after discussion at the consensus meeting. Five of these were in the social domain and were considered relevant and useful in the Malawian setting. These items are shown in Table S2. Socioeconomic status had a significant effect in the logistic regression analysis in up to 26% of items in some domains and nutritional status had a similar effect in the analysis and attainment of milestones in all developmental domains (HAZ score in 47%–65% of items and WAZ in 38%–56% of items).

**Table 2:** Number (%) of items in each domain of development that had poor goodness of fit and where gender, socioeconomic status, HAZ, or WAZ were significant effects in logistic regression.

Domain of Development	Poor Goodness of Fit on Logistic Regression	Gender	Socioeconomic Status	HAZ	WAZ
**Gross motor (** ***n*** ** = 34)**	0	1 (3%)	3 (9%)	17 (50%)	18 (52%)
**Fine motor (** ***n*** ** = 34)**	14 (41%)	2 (6%)	5 (15%)	18 (52%)	17 (50%)
**Language (** ***n*** ** = 34)**	19 (56%)	0 (0%)	7 (20%)	22 (65%)	19 (56%)
**Social (** ***n*** ** = 34)**	17 (50%)	5 (15%)	9 (26%)	16 (47%)	13 (38%)
**Total (** ***n*** ** = 136)**	50 (37%)	8 (6%)	24 (18%)	73 (54%)	67 (49%)

Figures 4–7 show the normal population reference ranges displayed as graphs of age ranges of attainment of milestones. There is one graph for each domain of development.

### Reliability

Overall, reliability was excellent (k>0.75) for 99% (134/136) of interobserver immediate reliability (Table 3), for 89% (121/136) interobserver delayed reliability, and 71% (96/136) of intra-observer–delayed 2-wk assessments. The remaining assessments had fair-to-very good reliability (k>0.4) with only two items having poor reliability (k<0.4) in the interobserver immediate category. In terms of the developmental domains, gross motor, fine motor, and social items had good kappa values for reliability, whereas in the language domain there were more moderate-to-good agreements. Delayed intra-observer reliability performed less well than the other forms of reliability in all the domains with excellent agreement in only 47%–88% of items, depending upon the domain.

**Table 3:** Reliability by area of development for final items in MDAT.

Domain of Development	Reliability	Kappa Score			Percentage Agreement		
		Excellent, >0.75	Fair to Good, 0.4–0.75	Poor, <0.4	>95%	85%–95%	<85%
**Gross motor (34)**	Interobserver immediate	34 (100%)	0	0	34 (100%)	0	0
	Interobserver delayed	34 (100%)	0	0	30 (88%)	4 (12%)	0
	Intra-observer delayed	27 (79%)	7 (21%)	0	12 (35%)	22 (65%)	1(3%)
**Fine motor (34)**	Interobserver immediate	34 (100%)	0	0	34 (100%)	0	0
	Interobserver delayed	30 (88%)	4 (12%)	0	27 (79%)	7 (21%)	0
	Intra-observer delayed	23 (68%)	11 (32%)	0	7(20.6%)	25 (74%)	2(6%)
**Language (34)**	Interobserver immediate	32 (94%)	0	2 (6%)	32 (94%)	2 (6%)	0
	Interobserver delayed	26(76%)	8 (24%)	0	6 (18%)	26 (76%)	2(6%)
	Intra-observer delayed	30 (88%)	4 (12%)	0	25 (74%)	8 (24%)	1(3%)
**Social (34)**	Interobserver immediate	34 (100%)	0	0	25 (74%)	9 (26%)	0
	Interobserver delayed	31 (91%)	3 (9%)	0	28 (82%)	5 (15%)	1(3%)
	Intra-observer delayed	16(47%)	18 (53%)	0	7(21%)	21(62%)	5(15%)
**Totals (136)**	Interobserver immediate	134 (99%)	0	2 (1%)	125 (92%)	11 (8%)	0
	Interobserver delayed	121 (89%)	15 (11%)	0	91(67%)	42(31%)	3(2%)
	Intra-observer delayed	96 (71%)	38(29%)	0	51(38%)	76 (56%)	9(6%)

### Final Developmental Tool after Consensus

After consensus, from the draft tool of 185 items, we created a final version of the tool with 136 items, 34 in each domain of development (see Figures S2–S5 for this final questionnaire). Items removed at consensus and the reasons for this are outlined in Table 4. In the gross motor domain, most items in the final tool (27/34) were retained or modified from the preliminary tool, whereas in the social domain, only 12/34 items remained from the preliminary version in their original or modified form, and 22/34 new items were created, most of these (18/24) being newly created from the qualitative study described elsewhere [12].

**Table 4:** Reasons for removal of items in the consensus meeting within each domain of development.

Domain of Development	Items Removed	Reason for Removal
**Gross motor**	Head erect continuously when sat up or held	Age range for item exactly the same as another item
	Stands alone for a few seconds	Age range for item exactly the same as another item
	Walks backwards	Poor fit^a^. Reported as “difficult to get children to do this.”
	Jumps with both feet off the ground	Age range for item exactly the same as another item
	Jumps over rope 10 cm off ground	Age range for item exactly the same as another item that involves jumping
	Throws one or more balls into a basket (out of five)	Poor interobserver and intra-observer reliability and poor fit (difficult to be consistent with item)
	Carries a cup on head with no hands	Sex specific, girl's task
**Fine motor**	Plays with cup and spoon in purposeful manner	Age range for item exactly the same as another item
	Tower of blocks up to eight blocks	Poor fit and age range exactly same as other item
	Can do “Sharp” (put thumb up in specific way)	Sex specific, boy's item.
	Is able to play Chipapa (clapping game)	Sex specific, girl's item.
	Able to fold paper in two halves	Not achieving 90% by 7 y. Found difficult for children to do by examiners.
	Makes a bridge with six bricks	Poor reliability. Not achieving 90% by 7 y
	Makes stairs with bricks	Poor reliability. Not achieving 90% by 7 y
	Copies row of bottle tops	Poor reliability. Not achieving 90% by 7 y
	Copies square pattern of bottle tops	Poor reliability. Not achieving 90% by 7 y
**Language**	Says four or more words	Poor fit. Not easy to be so exact.
	Says eight or more words	Poor fit. Not easy to be so exact.
	Identifies at least two objects	Poor fit.
	Uses many words >20	Poor fit
	Points to body parts: at least 1 body part	Age range for item exactly same as another item
	Names two objects	Poor fit.
	Knows his or her father's last name	Poor fit. Subjectively many children don't know father's last name
	Copies two lines of song well at home	Poor fit. Subjectively not clear item
	Number recall 1–4	Poor fit and age range same as another item
	Knows quantities	Poor reliability
	Sings two lines of song clearly	Poor fit and many missing
	Retells stories in brief manner	Poor fit and few achieving before 7 y
	Knows how old they are	Poor reliability. 90th centile not before 8 y
	Knows materials	Not achieving 90th centile by 7 y.
**Social**	Drinks from a cup but may spill some	Unclear question. Poor fit. Some normal children failing
	Wants to be escorted to pit latrine/toilet	Unclear what toilet is. Poor fit and reliability
	Able to imitate household chores	Poor logistic regression. Sex specific, girl's task
	Can do errands e.g., bring salt	Poor logistic regression. Sex specific, girl's task
	Able to play singing games	Sex specific, girl's task.
	Plays Masanje/house	Sex specific, girl's task.
	Spends more time with specific friend	Sex specific (boy's task) and poor fit
	Does housework properly useful round house	Not achieved by 7 y
	Knows how to take responsibility without being asked	Not achieved by 7 y

a Poor goodness of fit in logistic regression.

### Validity

The MDAT correctly identified almost all of the children with neurodisabilities, with 97% failing compared with 18% of normal age-matched controls. Sensitivity was therefore very high (97%), and specificity was 82%. When we compared the children's scores, those with neurodisabilities had average scores 63.9 points lower than age- and sex-matched controls, with highly significant differences in scores in all domains (Table 5).

**Table 5:** Comparison of scores for children with neurodisabilities or malnutrion and their age-matched controls using the MDAT.

Domain of Development	Children with ND (*n* = 80)	Normal Controls (*n* = 80)	*p*-Value	Sensitivity (95% CI)	Specificity (95% CI)	Children with WHZ <80% (*n* = 120)	Normal Controls (*n* = 120)	*p*-Value
**Gross motor**								
***n*** ** Passing** a **(%)**	4/80 (5%)	79/80 (99%)	<0.001	0.95 (0.87–0.98)	0.99 (0.93–0.99)	75/120 (63%)	119/120(99%)	<0.001
**Mean score (SD)**	**9.2 (6.9)**	**25.4 (6.1)**	<0.001	**—**	**—**	**16.5 (5.23)**	**20.7 (5.5)**	<0.001
**Fine motor**								
***n*** ** Passing** a **(%)**	4/72 (6%)	66/72 (92%)	<0.001	0.94 (0.86–0.98)	0.91 (0.82–0.96)	55/116 (47%)	109/116(94%)	<0.001
**Mean Score (SD)**	**7.9 (7.9)**	**25.2 (6.3)**	<0.001	**—**	**—**	**15.6 (6.9)**	**20.5 (5.8)**	<0.001
**Language**								
***n*** ** Passing** a **(%)**	11/66 (17%)	62/66 (95%)	<0.001	0.85 (0.74–0.92)	0.95 (0.87–0.99)	81/109 (74%)	109/109 (100%)	<0.001
**Mean score (SD)**	**7.4 (5.9)**	**22.7 (8.1)**	<0.001	**—**	**—**	**11.9 (5.2)**	**16.2 (6.4)**	**<0.001**
**Social**								
***n*** ** Passing** a **(%)**	8/71 (11%)	68/71 (96%)	<0.001	0.89 (0.79–0.95)	0.96 (0.88–0.99)	75/113 (66%)	111/113(98%)	<0.001
**Mean score (SD)**	**10.4 (7.5)**	**26.4 (7.1)**	<0.001	**—**	**—**	**18.4 (7.1)**	**20.2 (6.8)**	**0.107**
**All domains**								
***n*** ** passing** b **(%)**	2/79 (3%)	65/79 (82%)	<0.001	0.98 (0.93–0.99)	0.82 (0.73–0.91)	33/118 (28%)	111/118(94%)	<0.001
**Mean score (SD)**	**35 (20.4)**	**99.0 (25.9)**	<0.001	**—**	**—**	**62.5 (22.4)**	**77.4 (23)**	<0.001

CI, confidence interval; ND, neurodisability; WHZ (weight for height Z score).

^a^Passing in each domain = one or no failures in any one domain (gross motor, fine motor, language, social) for the age of the child.

b Passing for all domains = no failures in any domain of development.

When comparing the children with marasmus to controls, 72% failed the MDAT compared with 6% of controls. Children with marasmus had overall average scores 14.9 points lower than controls (Table 5), with scores significantly different in all domains except social development. Differences in scores were 5.1 points in fine motor but only 1.8 points in social development.

## Discussion

We have managed to develop a tool with normal reference values to assess childhood development up to the age of 6 y for a rural setting in Africa. We have demonstrated its sensitivity in the detection of neurodisability but also more subtle neurodevelopmental delay as seen in children with malnutrition. We have demonstrated good face and content validity of the tool. This instrument is therefore culturally appropriate for the rural sub-Saharan African setting of Malawi, and is likely to be applicable in other similar settings. The tool is easy to use, has good reliability, only requires a small basket of props, and takes approximately 30 min to administer. It also has clear pictorial representations of many of the items in the tool, making it understandable to all who use it. The MDAT could be used by local health workers with little training as well as by researchers needing a tool to use as an outcome measure when assessing development of children in these settings.

There is much evidence that the large scale problem of disability and developmental delay in resource-poor settings has a high total cost to societies and contributes to continuing cycles of poverty preventing improvements in children's achievement in these settings [1]. The benefits of preventative measures and integrated programmes to improve child development have been shown, however, few robust developmental tools are available to assess the outcome of these programmes [2]. The MDAT has demonstrated good sensitivity in detecting children with neurodisabilities as well the more subtle differences in development that would be expected between children with marasmus and normal age-matched controls [23]. To be able to use tools such as this to identify disability and developmental delay is an exciting prospect when there are few robust instruments for detection of disability, especially for those children under 2 y and where tools such as the “ten question disability screen” are inadequate [34].

We have been fortunate to have access to a large population of normal rural African children through antenatal clinics allowing us the opportunity to create normal reference values for a typical Malawian child population. The MDAT population is very similar in economic status to the Malawian childhood population. The percentages of children with stunting and malnutrition in the MDAT population were a little lower than those seen in the MDHS population, partly due to the fact that we excluded any children who were severely malnourished (<2 SD weight for height), but also because our population had more semi-urban children in it than the national average. We wanted a tool that reflected the normal population of Malawi, however, we also wanted to reflect a population that was clinically well. Although these conditions were difficult to achieve and the population used was not an “ideal” population (one in which health and development would be at its most ideal), it was a population that we felt reflected the normal population, but not including those with severe medical problems and in need of specific support.

Previous literature makes it clear that malnutrition will affect the achievement of developmental milestones [1],[35]. We have found that height for age and weight for age did affect the normal reference values in approximately half of the items in the tool, demonstrating that many of the developmental items are sensitive to differences in nutritional status between children. Furthermore, as expected, socioeconomic status within the groups studied does seem to also play a role in attainment of some items, particularly in the social domain. 85% of children in Malawi live in rural areas [18] with half of children stunted, therefore we would argue that a developmental tool should be appropriate for use in this type of population. The normal reference ranges have therefore not been adjusted for height for age, weight for age, or socioeconomic status.

We have developed a robust methodology for creating developmental assessment tools that can be applied in any setting and that could therefore be used in many different cultures worldwide. This includes a systematic series of initial qualitative studies, piloting, and translation to create a more culturally accessible tool that can then be tested and analysed item by item to attain reference values through logistic regression as well as to determine reliability. Before validation, a final consensus meeting with an appropriate group of assessors can select items for the final tool.

We have found in our construct validity studies that the MDAT is identifying 18% false positives. Our figures are, however, based on a case control method of sampling that may influence our results for sensitivity and specificity [36]. Although the tool is sensitive enough to pick up children with known neurodisabilities using the pass/fail scoring system that we have implemented, we still need to determine how well it can identify those with more subtle developmental delay. We have found that the MDAT can identify the developmental delay present in a subgroup of children with malnutrition. We identified 72% of children in this group with a delay in one or more areas of development and with average scores 14.9 points lower than the normal controls. This finding is consistent with evidence demonstrating that children with malnutrition have moderate developmental delay with overall DQ (developmental quotients) 20 to 30 points lower than normal children [23],[24],[35]. Despite these results, further research into scoring of the tool, as well as validation in groups of children with more subtle developmental delay, is necessary to provide further evidence of how the tool works.

The MDAT has broad applications both as a clinical tool in early identification of neurodevelopmental problems and as an outcome measure, for example in clinical trials of perinatal interventions. It is clear that settings such as Malawi have limited services to support this population and at present this tool may be more useful as an outcome measurement tool for research practice. However, by being able to identify children with neurodevelopmental delay, scarce government resources as well as international intervention programmes can be directed most effectively. Furthermore, without measures such as this, there will be no evidence as to whether interventions to improve outcomes in early childhood are effective in these settings.

## Supporting Information

Figure S1 Basket of items used in the MDAT.
(5.17 MB TIF)

Figure S2 Final MDAT questionnaire in four sections: (A) Gross motor.
(0.65 MB TIF)

Figure S3 Final MDAT questionnaire in four sections: (B) Fine motor.
(0.57 MB TIF)

Figure S4 Final MDAT questionnaire in four sections: (C) Language.
(0.51 MB TIF)

Figure S5 Final MDAT questionnaire in four sections: (D) Social.
(0.66 MB TIF)

Table S1 Numbers of children recruited in each age group for item testing and creation of normal reference ranges for the MDAT.
(0.07 MB DOC)

Table S2 Gender-specific items in the MDAT.
(0.03 MB DOC)

Text S1 MDAT instruction manual (in English and Chichewa).
(0.15 MB PDF)

## Abbreviations

HAZ: height for age
MDAT: Malawi Developmental Assessment Tool
MDHS: Malawi Demographic Health Survey
SD: standard deviation
WAZ: weight for age
