# Supplementary figures and images for: The Malawi Developmental Assessment Tool (MDAT): The Creation, Validation, and Reliability of a Tool to Assess Child Development in Rural African Settings
Source: PLoS Med. 2010 May 25;7(5):e1000273. doi: 10.1371/journal.pmed.1000273 (PMC2876049; doi:10.1371/journal.pmed.1000273)

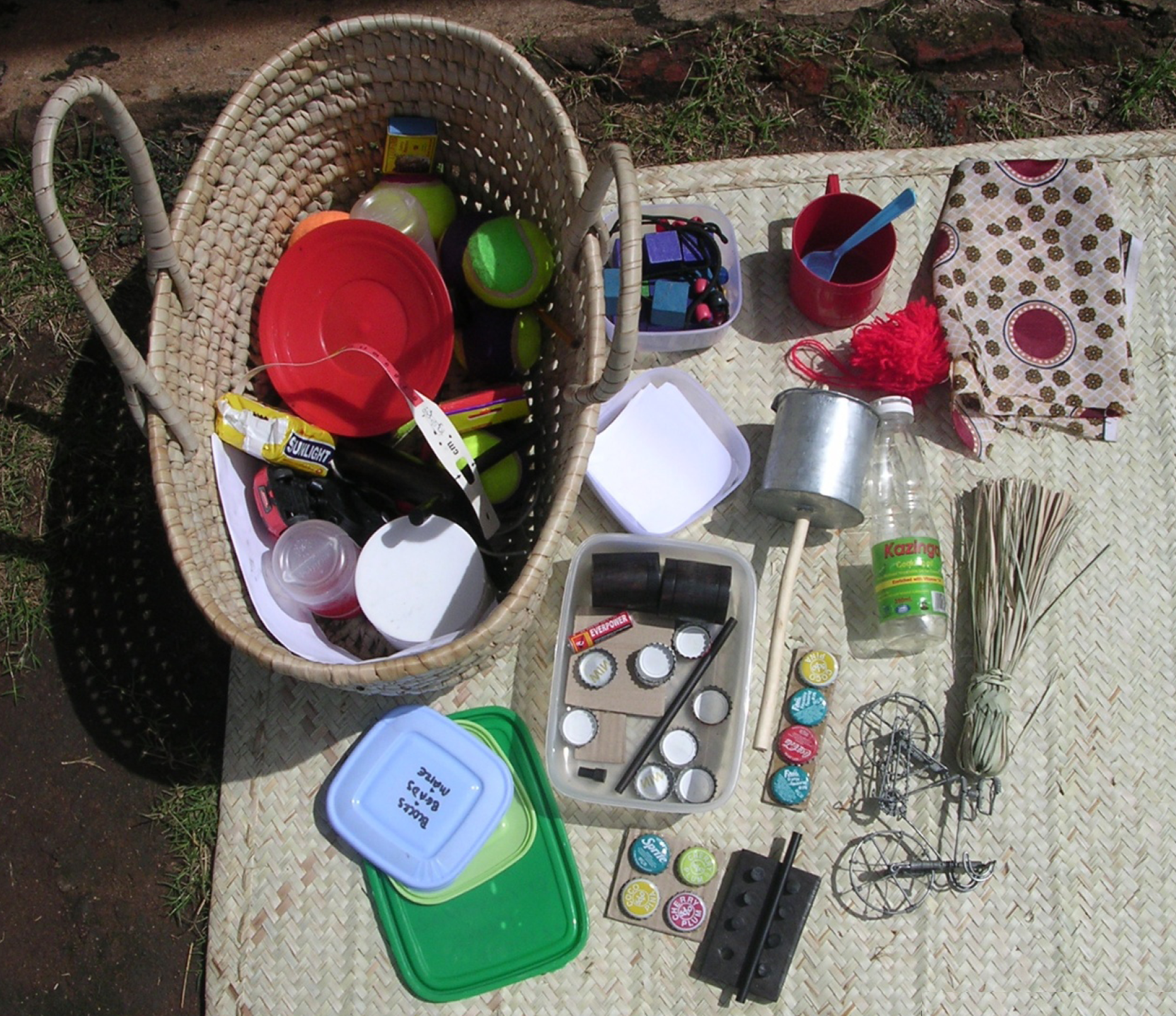

Supplement: Figure S1 — Basket of items used in the MDAT. (5.17 MB TIF) [file pmed.1000273.s001.tif]

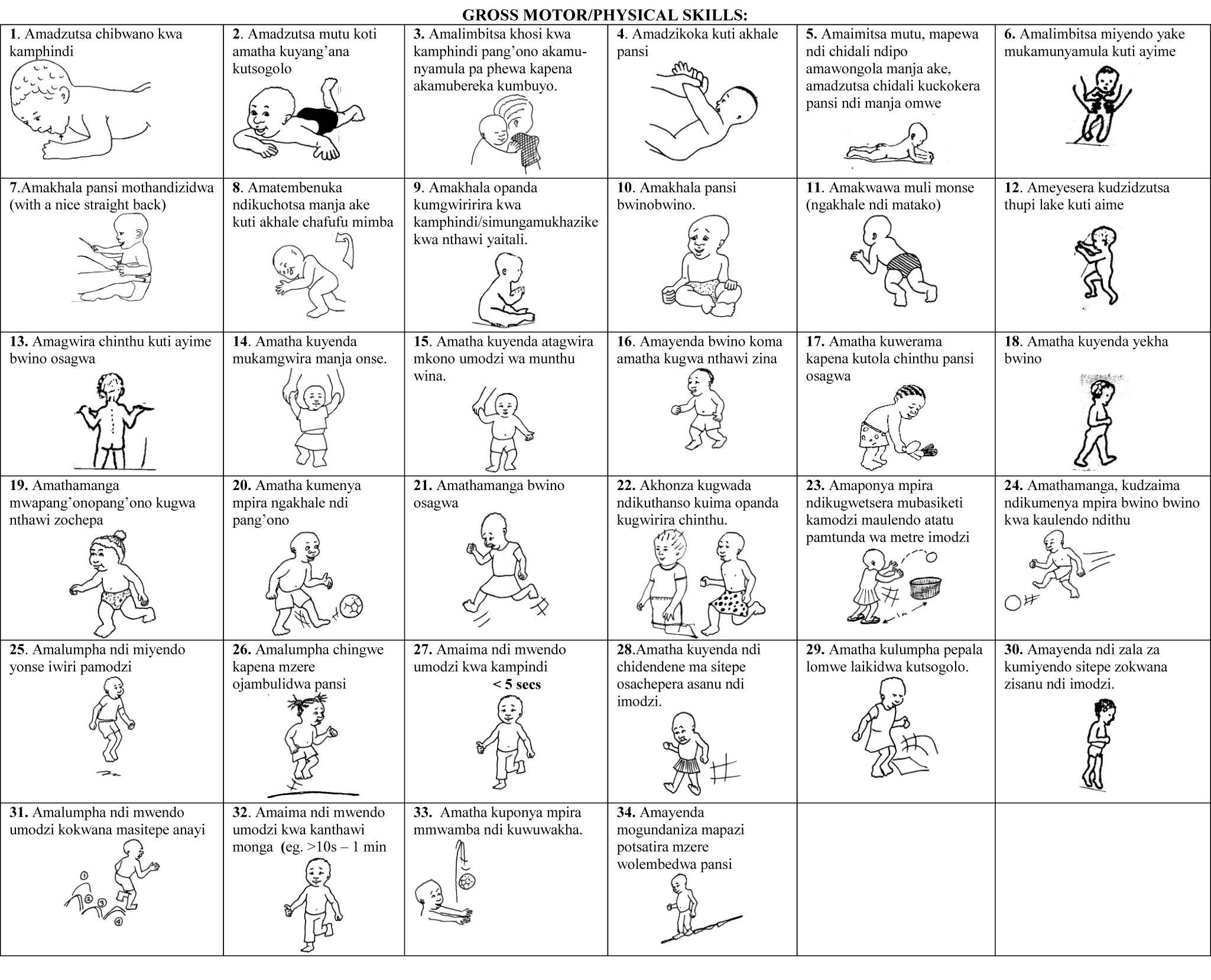

Supplement: Figure S2 — Final MDAT questionnaire in four sections: (A) Gross motor. (0.65 MB TIF) [file pmed.1000273.s002.tif]

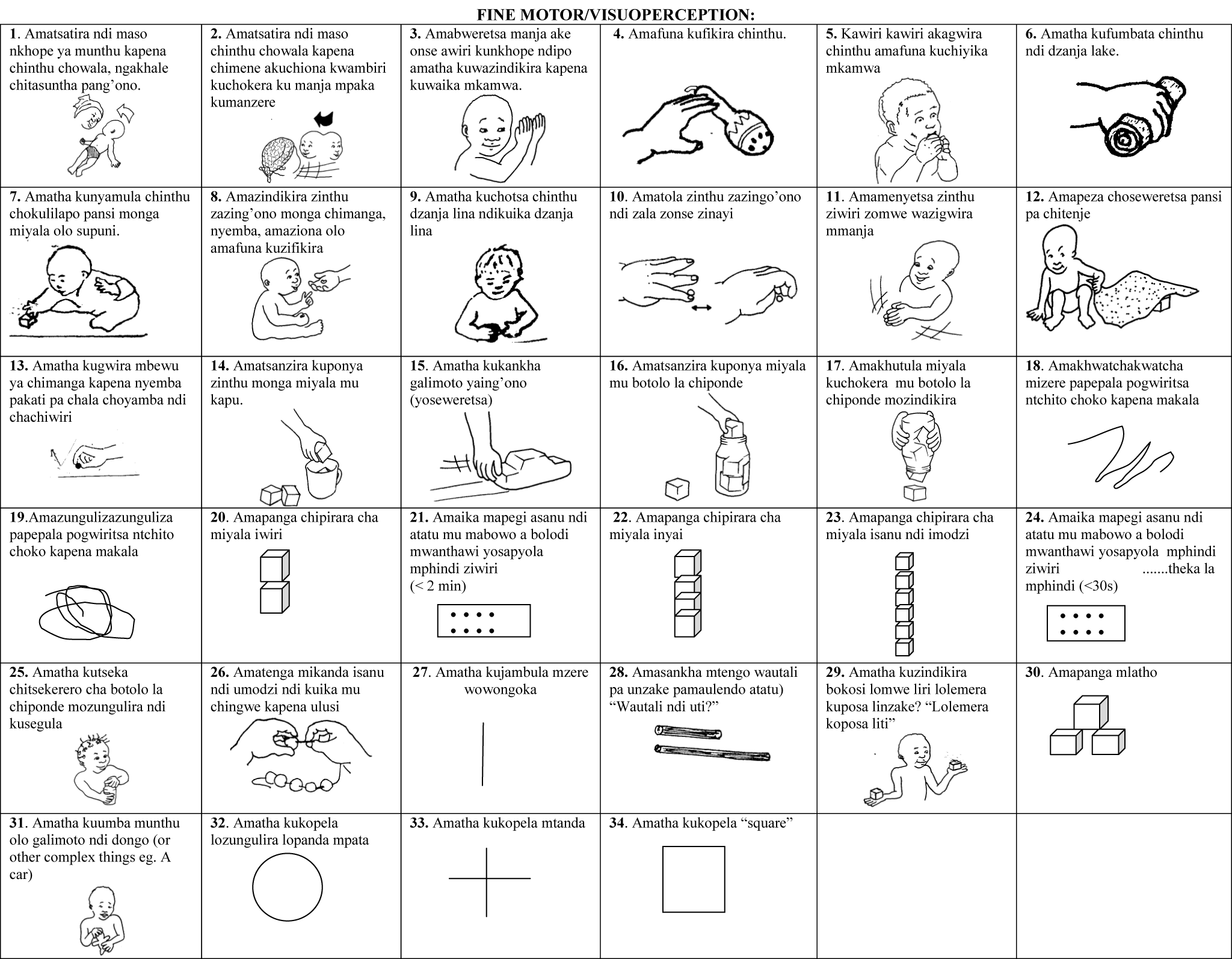

Supplement: Figure S3 — Final MDAT questionnaire in four sections: (B) Fine motor. (0.57 MB TIF) [file pmed.1000273.s003.tif]

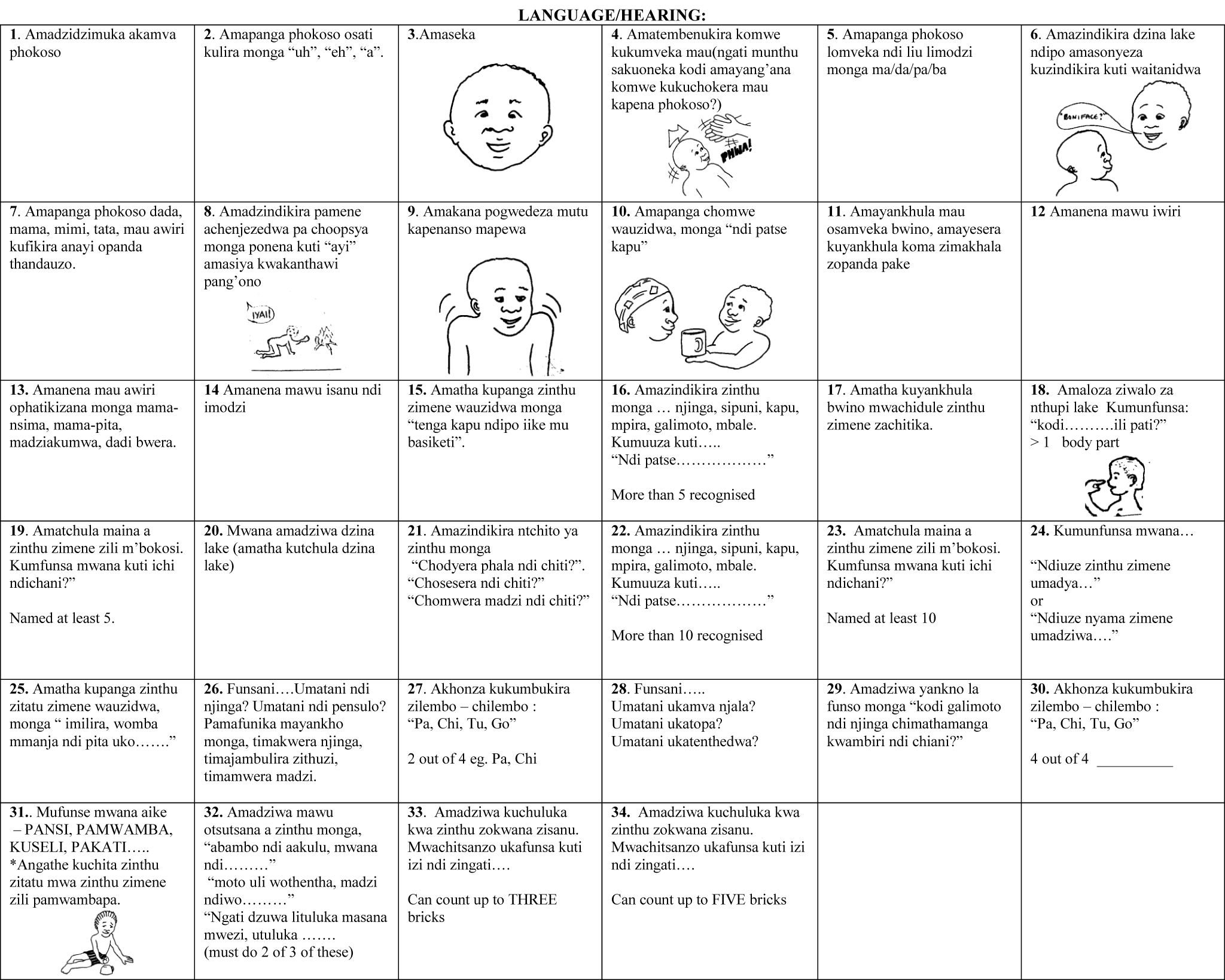

Supplement: Figure S4 — Final MDAT questionnaire in four sections: (C) Language. (0.51 MB TIF) [file pmed.1000273.s004.tif]

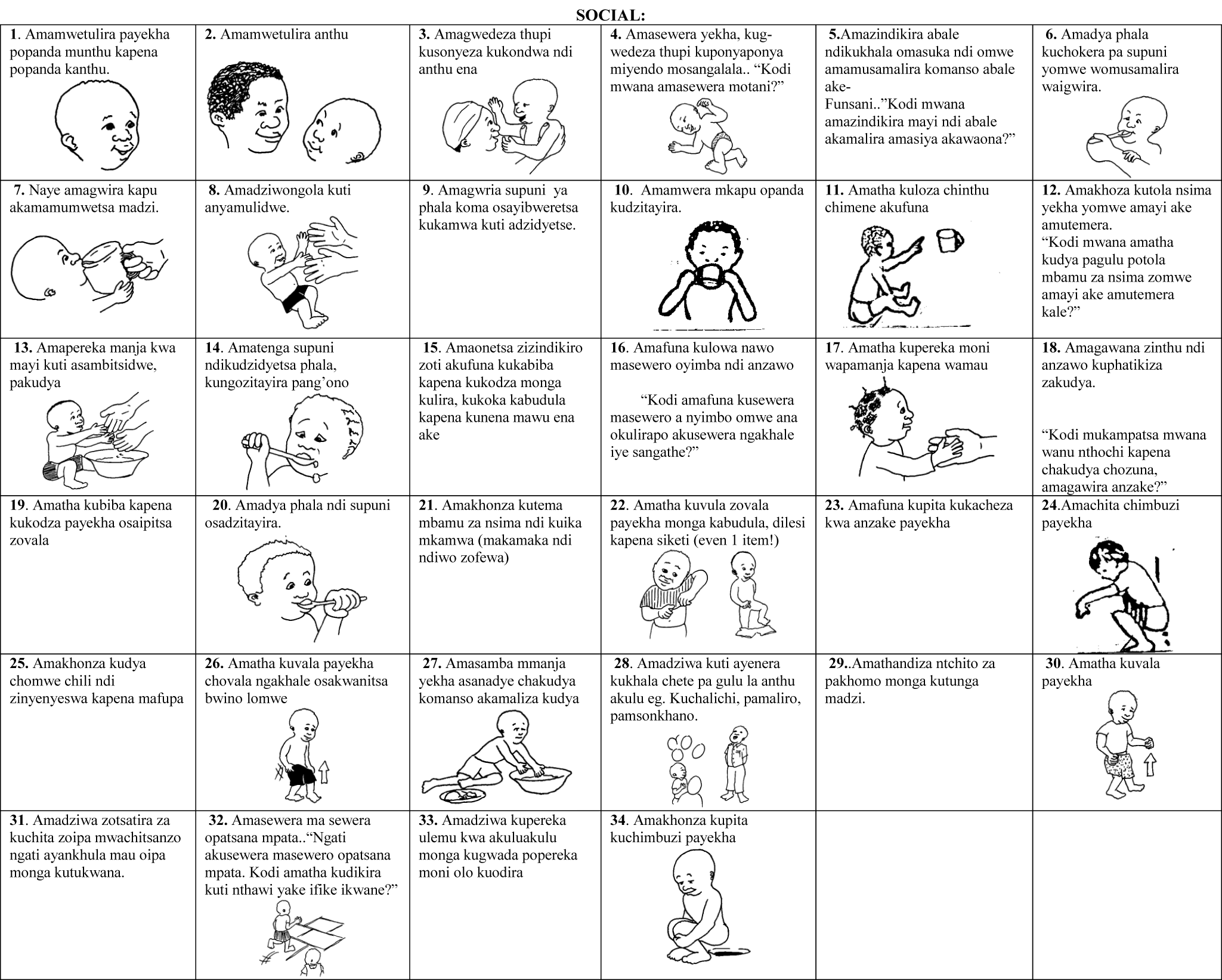

Supplement: Figure S5 — Final MDAT questionnaire in four sections: (D) Social. (0.66 MB TIF) [file pmed.1000273.s005.tif]
