# Supplementary material for: The Malawi Developmental Assessment Tool (MDAT): The Creation, Validation, and Reliability of a Tool to Assess Child Development in Rural African Settings
Source: PLoS Med. 2010 May 25;7(5):e1000273. doi: 10.1371/journal.pmed.1000273 (PMC2876049; doi:10.1371/journal.pmed.1000273)
Supplement: Table S1 — Numbers of children recruited in each age group for item testing and creation of normal reference ranges for the MDAT. (0.07 MB DOC) [file pmed.1000273.s006.doc]

| Age group | age range | Male | Female | Missing sex | Total |
| --- | --- | --- | --- | --- | --- |
| 1 | 0-1 mo | 51 | 37 | 2 | 90 |
| 2 | 1-2 mo | 76 | 55 | 4 | 135 |
| 3 | 2-3 mo | 35 | 19 | 3 | 57 |
| 4 | 3-4 mo | 37 | 29 | - | 66 |
| 5 | 4-5 mo | 27 | 24 | 1 | 52 |
| 6 | 5-6 mo | 20 | 23 | 2 | 45 |
| 7 | 6-7 mo | 21 | 12 | 3 | 36 |
| 8 | 7-8 mo | 23 | 27 | 3 | 53 |
| 9 | 8-9 mo | 18 | 25 | 2 | 45 |
| 10 | 9-10 mo | 17 | 17 | 1 | 35 |
| 11 | 10-11 mo | 19 | 24 | 1 | 44 |
| 12 | 11-12 mo | 10 | 16 | - | 26 |
| 13 | 12-13 mo | 19 | 18 | 1 | 38 |
| 14 | 13-14 mo | 12 | 10 | - | 22 |
| 15 | 14-15 mo | 11 | 16 | 1 | 28 |
| 16 | 15-16 mo | 12 | 12 | - | 24 |
| 17 | 16-17 mo | 9 | 12 | - | 21 |
| 18 | 17-18 mo | 14 | 11 | 1 | 26 |
| 19 | 18-19 mo | 11 | 11 | 3 | 25 |
| 20 | 19-20 mo | 9 | 10 | 1 | 20 |
| 21 | 20-21 mo | 5 | 11 | - | 16 |
| 22 | 21-22 mo | 10 | 14 | - | 24 |
| 23 | 22-23 mo | 10 | 7 | 1 | 18 |
| 24 | 23-24 mo | 10 | 7 | - | 17 |
| 25 | 24-30 mo | 32 | 28 | 2 | 62 |
| 26 | 30-36 mo | 30 | 36 | - | 66 |
| 27 | 36 -42mo | 19 | 29 | 2 | 50 |
| 28 | 42-48 mo | 17 | 39 | 1 | 57 |
| 29 | 48-54 mo | 16 | 33 | 1 | 50 |
| 30 | 54-60 mo | 21 | 32 | - | 53 |
| 31 | 60-66 mo | 26 | 20 | - | 46 |
| 32 | 66-72 mo | 15 | 23 | 1 | 39 |
| 33 | 72-78 mo | 23 | 27 | 3 | 53 |
| 34 | 78-84 mo | 4 | 3 | - | 7 |
|  | Total | 689 | 717 | 40 | 1446 |

**Supporting Table 1.** Numbers of children recruited in each age group for item testing and creation of normal reference ranges for the MDAT
