# Supplementary material for: The Malawi Developmental Assessment Tool (MDAT): The Creation, Validation, and Reliability of a Tool to Assess Child Development in Rural African Settings
Source: PLoS Med. 2010 May 25;7(5):e1000273. doi: 10.1371/journal.pmed.1000273 (PMC2876049; doi:10.1371/journal.pmed.1000273)
Supplement: Table S2 — Gender-specific items in the MDAT. (0.03 MB DOC) [file pmed.1000273.s007.doc]

| **Domain and item description** | **Consensus comment on gender effect** | **Plan for change** |
| --- | --- | --- |
| **GM:** Walks but falls over at times | No reason for sex specificity. May be due to wording of item. | Item to be re-worded slightly. |
| **FM:** Picks longest stick | No reason for sex specificity. Item not sex specific on preliminary study. | No change to item. |
| **FM:** Copies a circle with chalk | No reason for sex specificity. Item not sex specific on preliminary study. | No change to item. |
| **Social:** Recognises or settles with caregivers | No reason for sex specificity. | No change to item. |
| **Social:** Can hold a spoon and take phala by self | No reason for sex specificity. | No change to item. |
| **Social:** Wants to go and visit a friend’s house | No reason for sex specificity. Item not clear as to whether child could visit friend or relative and whether visit could be in same compound. | Change wording to say “can be in same compound”. Have also made clearer instructions. |
| **Social:** Does household chores or helps in useful way | Not clear in item what the child using to draw water as will affect item attainment. “Should probably keep as bridges a gap” | Put in extra examples for boys. |

**Supporting Table 2.** Gender specific items retained in the MDAT
