# Supplementary material for: The Malawi Developmental Assessment Tool (MDAT): The Creation, Validation, and Reliability of a Tool to Assess Child Development in Rural African Settings
Source: PLoS Med. 2010 May 25;7(5):e1000273. doi: 10.1371/journal.pmed.1000273 (PMC2876049; doi:10.1371/journal.pmed.1000273)
Supplement: Text S1 — MDAT instruction manual (in English and Chichewa). (0.15 MB PDF) [file pmed.1000273.s008.pdf]

MALAWI DEVELOPMENTAL ASSESSMENT TOOL

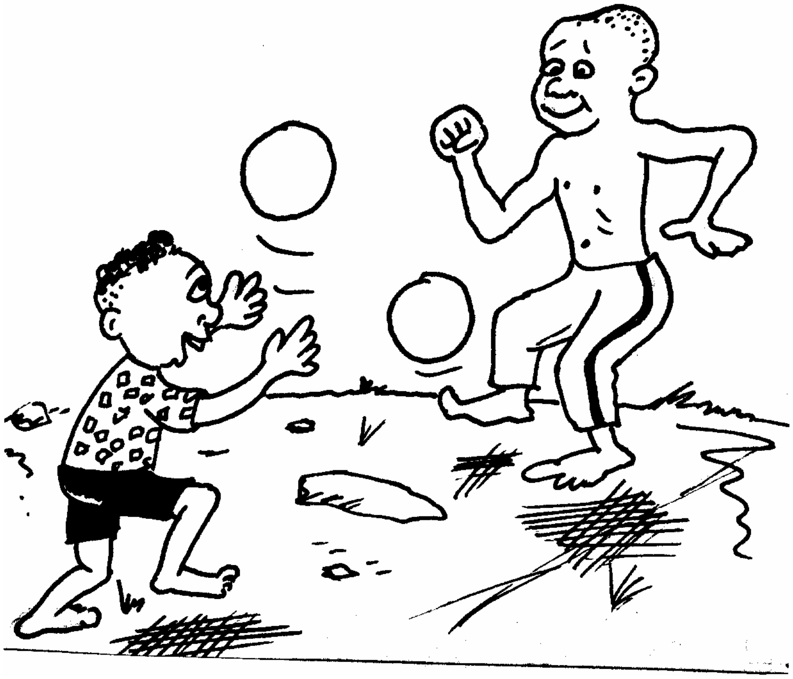

INSTRUCTION BOOK

© MDAT Research Team

CONTENTS:

ITINERARY OF ITEMS IN KIT……………………. Page 3

GROSS MOTOR questions………………………… Page 4 - 6

FINE MOTOR questions…………………………… Page 7 - 10.

LANGUAGE questions……………………………… Page 11 - 14

SOCIAL questions…………………………………… Page 15 - 17

Developmental kit (Itinerary)

1. 5 Balls (tennis ball size)
2. Naming objects (10 of them)
i. Broom (copy of larger one)
ii. Matchbox
iii. Plastic bottle e.g. for oil
iv. Plate (plastic from market)
v. Cup (plastic from market)
vi. Spoon (plastic from market)
vii. Soap
viii. Pencil or ball point pen
ix. Coin (any type)
x. Bicycle made out of wire
3. Blocks – 12 (square one inch size)
4. Chiponde (peanut butter) bottle or other plastic bottle with easy screw top
5. Maize pieces (dried)
6. Sound tin/rattle
7. Plain paper or other such thing to write on with chalk/charcoal
8. Wooden containers looking the same but of different weights (one hollow and one with sand)
9. Sticks of two different lengths
10. Red woollen ball (pompom – can be hand-made)
11. Board with eight pegs to put in.
12. Chalk
13. Basket (for putting items in and for throwing balls into)

14. Chitenje material (cloth)
15. Car (made of wood or plastic one from local market)
16. Beads (found in market as used to put in hair)
17. String 1 metre long

GROSS MOTOR:

1. **Lifts chin up off the floor for a few seconds:** (Amadzutsa chibwano kwa kamphindi) Put the child on their stomach on a flat surface or mat. See if the child can at least lift his/her head so that the chin is off the surface for a short period of time.

2. **Prone (on his or her tummy), can lift head up to 90 degrees:** (Amadzutsa mutu koti amatha kuyang'ana kutsogolo) Put child on his/her stomach on a flat mat. See if the child is able to lift head and chest up so that his or her face makes a 90 degree angle with the mat for at least several seconds. The child may support themselves on their forearms.

3. **Holds head upright for a few seconds:** (Amalimbitsa khosi kwa kamphindi pang'ono. Akamunyamula pa phewa kapena kumbuyo.) Hold the child in the sitting position or on mum's shoulders. See if child is able to hold his or her head upright and steady for at least several seconds without the head moving around too much all the time.

4. **Pulls to sit with no head lag:** (Amadzikoka kuti akhale pansi) Put the child on his or her back on the mat. Hold the child's hands and pull the baby up to a sitting position. See if the child's head comes with the body and does not lag or fall behind at any time while the body is being pulled up. If it does from early on, do not keep on trying.

5. **Lifts head, shoulders and chest when prone.** (Amaimitsa mutu, mapewa ndi chidali ndipo amawongola manja ake, amadzutsa chidali kuchokera pansi ndi manja omwe ndi kuwaongola) Put the child on their stomach on a flat surface. See if the child is able to lift their head and chest off the surface using their arms for support so that he or she is looking ahead of themselves or even upwards.

6. **Bears weight on legs (holds legs strongly when put in standing position):** (Amalimbitsa miyendo yake mukamunyamula kuti ayime) Hold the child up as if they were standing position with their standing on the table. See whether the child supports his or her weight on the legs for a few seconds if you let your support go a little bit.

7. **Sits with help.** (amakhala pansi mothandizidwa) Put the child in the sitting position either on mum's lap or on the floor in between mum's legs. See if the child will sit with the help of mum with a nice straight back.

8. **Rolls over from back to front:** (Amatembenuka ndikuchotsa manja ake kuti akhale chafufu mimba) See if the child rolls over any time during the examination, otherwise ask them mother whether she has observed this. Can put child in prone position and can show

the baby a toy and then pull it upwards beyond child's sight. The child may roll over to try and grab the toy or just because it wants to continue to look at it.

9. **Sits without help for a period of time but unable to be left for a long length of time.** (Amakhala opanda kumgwiririra kwa kamphindi/simungamukhazike kwa nthawi yaitali) The mother can not yet leave the child sitting alone for any length of time, but he or she is able to sit for a short time unsupported with pillows etc (a matter of seconds).

10. **Sits by self well:** (Amakhala pansi bwinobwino) Can be left sitting on the floor with toys to play with and can maintain balance in this position by self for a long period of time.

11. **Crawls (in any way):** (Amakwawa muli monse ngakhale ndi matako) Is able to get about by either shuffling on bottom or on all four limbs.

12. **Pulls self to stand/ trying to get to standing:** (Amayesera kudzutsa thupi lake kuti aime) Will grasp hold of furniture or other objects and pull self to standing position. Does not need to stay there for any length of time and may fall back down quite quickly.

13. **Able to stand well if holding on to things:** "Uses an object with the purpose of getting to standing" (Amagwira chinthu kuti ayime bwino osagwa) See if the child can stand and hold on to a solid object such as the side of a khonde or a chair for a few seconds. May pass this without having got themselves to the standing position.

14. **Walks using both hands of somebody:** (Amatha kuyenda mukamgwira manja onse) If you hold both hands to balance the baby, can they take several steps without tripping or falling?

15. **Walks with help (using somebody's hand as if led or a piece of furniture):** (Amatha kuyenda atagwira mkono umodzi wa munthu wina) See if child can walk with the help of the mother holding out one hand to the child. See if the child will take a few steps with help or on the furniture, but not alone.

16. **Walks, but falls over at times:** Amayenda bwino koma amatha kugwa nthawi zina) Is able to walk but not that confidently yet and falls over at times/quite often still.

17. **Stoops over and gets back up e.g.** Picks up object off the floor without falling (Amatha kuwerama kapena kutola chinthu pansi osagwa) See if the child is able to bend over and pick up a toy and return to a standing position without holding on to things around them or sitting down.

18. **Walks well.** (Amatha kuyenda yekha bwino) See if child walks well without falling over often with good balance.

19. **Runs, but basic running – may fall over at times.** (Amathamanga mwapang'ono pang'ono kugwa nthawi zochepa) Able to get about quickly but not completely confident and falls over occasionally. Children often run with their legs side to side in an unstable fashion.

20. **Kicks a ball in any way/tries to kick a ball:** (Amatha kumenya mpira ngakhale ndi pang'ono) Can kick a ball by trying to move leg forward to meet it or by walking into it. Not necessarily kicking well with a really good swing of the leg.

21. **Runs well (confidently), and can stop and start without falling over:** (Amathamanga bwino osagwa) Runs with confidence with feet lifted up behind him or her as legs go forward.

22. **Kneels (as in a respectful way) and gets up without using hands:** (Akhonza kugwada ndikuthanso kuima opanda kugwirira chinthu) Able to get down on to his knees staying in an upright position and then get up without using hands.

23. **Throws a ball into a basket (at least one of 3 times) 1 metre away**: (Amaponya mpira ndikugwetsera mubasiketi kamodzi maulendo atatu pamtunda wa metre imodzi) Stands **one metre** away from examiner's basket and is able to throw the ball in on at least one of three occasions when it is tried. Demonstrated by examiner first. Can use the metre long string to measure this.

24. **Runs, stops and is able to kick a ball some distance:** (Amathamanga, kudzaima ndikumenya mpira bwino bwino kwa kaulendo ndithu) Able to run up to the ball and kick it well a good distance with a good swing forward of the leg and balancing on one foot.

25. **Jumps with feet together off the ground:** (Amalumpha ndi miyendo yonse iwiri pamodzi) Able to jump with both feet leaving the floor together. Needs to get both feet off the ground.

26. **Jumps over line/string on the ground:** (Amalumpha chingwe kapena mzere ojambulidwa pansi) Able to jump well lifting both feet off the ground together over a string/line painted on the ground. Not a hop or skip. Feet should remain together and both feet reaching the floor at the same moment.

27. **Stands on one foot for less than 5 seconds:** (Amaima ndi mwendo umodzi kwa kampindi) Ask child to raise one foot usually by bending his knee and maintain a good balance on his other foot for at least a second – up to 5 seconds

28. **Walks on heels for 6 + steps:** Can walk 6 steps on heels easily. (Amatha kuyenda ndi chidendene ma sitepe osachepera asanu ndi imodzi.

29. **Jumps over a piece of paper (widthways):** Amalumpha pepala lomwe laikidwa kutsogolo) Put a piece of the examination paper (A4 size) on the ground. Ask the child to stand close to the side of the paper with both feet together and to jump over the less wide part of the paper and landing with both feet together. Can be demonstrated. Both feet need to come off the ground.

30. **Walks on tip toes for six or more steps:** (Amayenda ndi zala za kumiyendo sitepe zokwana zisanu ndi imodzi) Able to balance on tip toes and walk 6 steps keeping up on the toes well with good balance.

31. **Hops on one foot without support, has to go four steps:** (Amalumpha ndi mwendo umodzi kokwana masitepe anayi) Makes three consecutive hops with the same leg raised throughout. Can be demonstrated by the examiner first.

32. **Stands on one foot for a longer time – (at least 5 seconds and up to a minute)** (Amaima ndi mwendo umodzi kwa kanthawi monga.) Ask child to raise one foot usually by bending his knee and maintain a good balance on his other foot for more than 5-10 seconds, up to a minute or two counts.

33. **Can throw a ball up in the air and catch it with 2 hands.** (Amatha kuponya mpira mmwamba ndi kuwuwakha.) Can throw the ball up a good distance and catch it with two hands together.

34. **Heel/toe walk with one foot behind the other along the string with good balance:** (Amayenda mogundaniza mapazi potsatira mzere wolembedwa pansi) Can walk along the same line but this time heel to toe with his feet touching with good balance.

FINE MOTOR & PERFORMANCE:

1. **Follows mother's or guardian's face/object to the midline: (**Amatsatira ndi maso nkhope ya munthu kapena chinthu chowala, ngakhale chitasuntha pang'ono) Put the child on his/her back and hold the red yarn above the child's face at about 15 cms. Move the yarn in an arc from one side to the other. See if the child follows the yarn with eyes to the midline/ a short distance.

2. **Follows object or fixes and follows on face or bright object (red pompom) with eyes through 180 degrees.** (Amatsatira ndi maso chinthu chowala kapena chimene akuchiona kwambiri kuchokera ku manja mpaka kumanzere) See if the child will follow the face or bright red object through a complete arc from left to right e.g. through 180 degrees.

3. **Puts hands together/awareness of hands/puts in front of eyes/mouth:** (Amabweretsa manja kunkhope ndipo amatha kuwazindikira kapena kuwaika mkamwa) While the child is lying on his or her back, see if the hands are brought together at the midline of the body and put them in front of his eyes to look at them. . Do not cradle the baby in the parent's arms during this,

4. **Reaches out for a large thing eg. Rattle or red yarn :** (Amafuna kufikira chinthu) If child is sitting in parent's lap, see if the child will try and grasp an object such as the red yarn or the rattle. Examiner to bring the rattle or red yarn near the child and observe whether child could reach object. Use bright object that child can see well.

5. **When holding objects, tends to put them in mouth:** (Kawiri kawiri akagwira chinthu amafuna kuchiyika mkamwa) Often when the baby has picked up toys, they like to explore them by putting them in their mouth.

6. **Grasps hold of a large thing e.g. Handle of the rattle or plastic spoon:** (Amatha kufumbata chinthu ndi dzanja lake) See if the child is then able to pick up/grasp the object that he/she has been reaching for. The examiner gives the child a spoon, rattle or a pen in his hands and saw whether the child could grasp the object in his hands without letting it fall off.

7. **Can pick up a larger object from the ground e.g.** The spoon or the rattle or a block (Amatha kunyamula chinthu chokulilapo pansi monga miyala olo supuni.) When sitting on mum's lap or on the floor, likes to try and reach out and pick up objects off the ground or table that are interesting. Keep objects slightly out of reach, so that they have to reach for them.

8. **Can see a small object such as a piece of maize or a bean –** can see it and reach for it (Amazindikira zinthu zazing'ono monga chimanga, nyemba, amaziona olo amafuna kuzifikira) When the child is sitting on mum's lap, place a small object like a piece of maize or a bean in your hand and show it to the child. See if they notice it by pointing, looking at it, or if they want to touch it or pick it up. They don't have to pick it up – can just look at it.

9. **Transfers objects from one hand to another hand.** (Amatha kuchotsa chinthu dzanja lina ndikuika dzanja lina) Give the child a block and then try and give them another to the same hand. The child will often pass the first block to the other hand so that he or she can take the second one. Always use blocks, not other objects.

10. **Picks up small things with all four fingers in a RAKING fashion**: (Amatola zinthu zazing'ono ndi zala zonse zinayi)

11. **Strikes on object with another in imitation with the examiner.** (Amamenyetsa zinthu ziwiri zomwe wazigwira mmanja)If the child holds one block in each hand and hits the blocks together or if he strikes any two objects together. Usually done with one block in each hand. Not with pots and pans and lids which are larger. Needs to be SEEN not just asked….Examiner takes a block in each hand and strikes them while the child is watching. The child was given a block in each hand and was told to do the same. The examiner repeated the procedure till the child could imitate properly.

12. **Finds object under the chitenje (piece of cloth):** (Amapeza choseweretsa pansi pa chitenje) Put an interesting object eg. Car or rattle, under a piece of cloth while the child is watching and see if they then realise where it has been hidden and look for it.

13. **Neat pincer grasp, picks up maize or bean with thumb and one finger:** (Amatha kugwira mbewu ya chimanga kapena nyemba pakati pa chala choyamba ndi chachiriwiri): If child is able to pick up maize with thumb and one of small fingers only.

14. **Puts blocks in and out of cup in imitation** (Amatsanzira kuponya zinthu monga miyala mu kapu) The child puts at least one cube in and out of a cup when shown by an examiner.

15. **Pushes a little car along** (Amatha kukankha galimoto yaing'ono (yoseweretsa)) Pushes the little car along playing with it and showing that he or she knows it should be moving.

16. **Puts blocks into chiponde bottle (short plastic bottle with screw on lid) in imitation** (Amatsanzira kuponya miyala mu botolo la chiponde) Puts at least one block into the chiponde bottle when shown by the examiner.

17. **Dumps blocks out of Chiponde bottle (short plastic bottle with lid) purposefully** (Amakhutula miyala kuchokera mu botolo la chiponde mozindikira) Show child on a couple of occasions how to dump the blocks out of the bottle. Then ask the child to get it out. Does not pass if child pulls blocks out with fingers or hands.

18. **Scribbles on paper (straight scribble)** : (Amakhwatchakhwatcha mizere papepala pogwiritsa ntchito choko kapena makala) The child must make marks on the paper, more than just a slight mark on the paper and this is usually in a back and forwards manner.

19. **Scribbles on paper (circular scribble)** Amazungulizazunguliza papepala pogwiritsa ntchito choko kapena makala) Scribbles but in a circular way not just back and forth.

20. **Tower of 2 blocks** (Amapanga chipirara cha miyala iwiri) Build a tower of two bricks, preferably of the same colour and see if the child will copy you. Count how many you get up to answer next items.

21. **Puts pegs into board in up to 2 minutes** (Amaika mapegi asanu ndi atatu mu mabowo a bolodi mwanthawi yosapyola mphindi ziwiri) Using the pegboards, take out all the pegs and see if the child can put them back in and see how many seconds it takes. They are allowed up to 2 minutes.

22. **Tower of 4 blocks** (Amapanga chipirara cha miyala inyai) Build a tower of four bricks, preferably of the same colour and see if the child will copy you. Count how many you get up to answer next items.

23. **Tower of 6 blocks** (Amapanga chipirara cha miyala isanu ndi imodzi) Build a tower of six bricks, preferably of the same colour and see if the child will copy you. Count how many you get up to answer next items.

24. **Puts pegs into board in up to 30 secs**. (Amaika mapegi asanu ndi atatu mu mabowo a bolodi mwanthawi yosapyola theka la mphindi……. Using the pegboards, take out all the pegs and see if the child can put them back in under 30 secs.

25. **Unscrews and screws back on the cap of the Chiponde bottle** (Amatha kutseka chitsekerero cha botolo la chiponde mozungulira ndi kutsegula) Is able to actually screw and unscrew the lid off and on the chiponde bottle, not just pulling it off.

26. **Can put 6 hair beads on to a shoe lace (thread them on)** (Amatenga mikanda isanu ndi ndi kuika mu chingwe kapena ulusi) See if the child is able to thread 6 hair beads on to a thread.

27. **Copies a vertical line (as drawn by the examiner) with charcoal/chalk within 30 degrees** (Amatha kujambula mzere wowongoka) Is able to draw a line on the paper or on the khonde with chalk that is similar to your vertical line by at least 30 degrees.

28. **Picks longest stick 3 of 3** (Amasankha mtengo wautali pa unzake pamaulendo atatu) "Wautali ndi uti?" Put down 2 sticks of different length on the ground and ask the child "which one is longest?" Do not indicate whether the child has done the correct response, but put them down again and ask the same question. See if the child can do it 3 times.

29. **Picks heaviest box 3 of 3 – is the child able to tell you which box is the heaviest?** (**Amatha kuzindikira bokosi lomwe liri lolemera kuposa linzake?)** "Lolemera koposa ndi liti?" Put the two weights in the child's hands and ask "which one is heavier?" Without indicating that that was the correct response, get the child to do it again two more times.

30. **Can make a bridge with bricks:** (Amapanga mlatho) Make a bridge for the child out of three bricks and see if the child can do the same. Keep yours up while the child is doing it and see if the child will copy you.

31. **Makes a doll or complicated car out of clay** (Amatha kuumba munthu olo galimoto ndi dongo) Can make a doll out of clay by themselves or another complicated toy such as a car.

32. **Copies a circle (needs to be complete) with chalk or in the sand with a stick** (Amatha kukopela lozungulira lopanda mpata) Show the child a circle and see if she can copy you. Can give up to 3 trials. Can do anything that is near to a circle that is complete or nearly complete. Continuous spiral motions as in scribbling do not count.

33. **Copies a cross with chalk** (Amatha kukopela mtanda) Draw a cross with chalk and see if the child can do the same. Anything where the two lines intersect counts. The two lines do not need to be the same size and can be at any angle. Can be in the sand with a stick or with a chalk or charcoal on paper or other surface. Can use back of questionnaire.

34. **Can draw a square:** (Amatha kukopela "square") Must have four sides and be a similar shape to a square but does not need to be perfect. Can be in the sand with a stick or with a chalk or charcoal on paper or other surface. Can use back of questionnaire.

LANGUAGE/HEARING:

1. **Startles or jumps/responds to sounds**: (Amadzidzimuka akamva phokoso) If child responds or jumps when a loud sound is made. Can be a response in any way e.g. Change in activity or expression or eye movement. Can ask mum whether the child starts at sudden noises in the house.

2. **Happy vocalising or making sounds – not crying**: (Amapanga phokoso osati kulira monga “uh”, “eh”, “a”.) Makes sounds other than crying eg. Throaty sounds or sounds such as “uh” or “eh” or “a, a, a” or gurgling sounds. Any vocal sound rather than crying.

3. **Laughs/chuckles:** (Amaseka) See if the child laughs out loud or whether the mum or guardian says they do.

4. **Turns to voice – if you are out of sight, does she/he look in the direction of your voice or sound?**: (Amatembenukira komwe kukumveka mau – ngati munthu sakuoneka kodi amayang’ana komwe kukuchokera mau kapena phokoso?) In reponse to the rattle or to mum’s voice, does the child turn his or her head towards the sound? Best to test both sides

5. **Uses single syllables or sounds, for example Ma, Pa, Da, Ba.** (Amapanga phokoso ndi liu limodzi monga ma/da/pa/ba) Makes any kinds of single syllable sounds as described.

6. **Responds to his or her name, turns and looks at you.** (Amazindikira dzina lake ndipo amasonyeza kuzindikira kuti waitanidwa) Is able to look up when asked for example “Chikondi?”. Be careful to distinguish between a response just to being called in a certain tone of voice and knowing his name. Does not need to say his or her name.

7. **Uses 2/4 syllable babble such as dada, mama, mimi, tata, but not specifically at anything or any person:** (Amapanga phokoso dada, mama, mimi, tata, mau awiri kufikira anayi opanda tanthauzo) The baby is able to make sounds that could be copied and sound almost like words. Needs to be clear or distinct sounds, not just vocalisations.

8. **Understands when being cautioned about danger, for example when saying “no” to child, they stop even briefly:** (Amazindikira pamene achenjezedwa pa choopsya monga

ponena kuti “ayi”amasiya kwakanthawi pang’ono) ‘Understands to stop doing something if the mother or guardian has said “no”. eg. Going too close to the fire.

9. **Indicates by gesture to say “No”:** (Amakana pogwedeza mutu kapenanso mapewa) Definate shaking of head or shrugging of shoulders in showing refusal. Not just turning away from the situation or withdrawing. Can ask mother if he does it at home if not seen.

10. **Follows simple commands (1 stage) eg.** “give me the cup”: (Amapanga chomwe wauzidwa, monga “ndi patse kapu”) Is able to understand when asked to give the examiner something. The examiner must be careful not to indicate by pointing that they want the object, they must only say it.

11. **Unclear talk/jabber in sentences - pretends to talk but does not actually make sense.** (Amayankhula mau osamveka bwino, amayesera kuyankhula koma zimakhala zopanda pake): May sit and talk to themselves e.g. When playing with things, but may not make sense. Sounds like proper words and sentences but does not make sense.

12. **Says 2 words, but words other than mama/dada:** (Amanena mawu angati?) Ask the parent how many words the child says. See if you can find out what they are. Can include names of people as long as they are directed. Mama or Dada said not at anyone in particular does not count. Words need to be directed at some particular thing. Answer yes here if two words or more.

13. **Says 2 words together:** (Amanena mau awiri ophatikizana monga mama-nsima, mama-pita, madziakumwa, dadi bwera): Note that the child is putting two words together in a meaningful phrase that indicates something. Examples are things like “mummy cup” or “water cup”.

14. **Says 6 words (words other than mama/dada):** (Amanena mawu angati?) Ask the parent how many words the child says. See if you can find out what they are. Can include names of people as long as they are directed. Mama or Dada said not at anyone in particular does not count. Words need to be directed at some particular thing. Answer “yes” here if more than six (6) words.

15. **Follows 2 stage commands e.g. “Go and take the cup and put some water in it”, “go and get the cup and put it in the basket”** (Amatha kupanga zinthu zimene wauzidwa monga “tenga kapu ndipo iike mu basiketi”) Is able to do a command where they have to understand and then do a succession of two things.

16. **Identifies objects in the basket – at least 5.** Bicycle, spoon, cup, ball, car, bottle, chitenje, little broom, plate, pencil.) Amazindikira zinthu monga... njinga, sipuni, kapu, mpira, galimoto, mbale. Kumuuza kuti…..) “Ndi patse……………” Count how many out of 10 the child has done. Make sure all 10 objects are put out. Answer YES if child can identify at least 5 of the ten objects. Doesn’t matter which ones.

17. **Speaks clearly in sentences. Child’s speech is fully understandable: (**Amatha kuyankhula bwino mwachidule zinthu zimene zachitika**)** Is the child able to narrate a story? Explain to mother “if you have sent your child somewhere can they recount just a little something of what happened e.g. Someone came looking for you”.

18. **Points to body parts:** > 1 part. (Amaloza ziwalo za mthupi lake. Kumufunsa:

"kodi……….ili pati?") Knows where the own child's body parts are eg. "Where is your nose? Where are your eyes? Where are your ears? Where is your mouth?" Answer YES if knows TWO or more body parts.

19. **Names 5 objects in the basket: (**Amatchula maina a zinthu zimene zili m'bokosi. Kumfunsa mwana kuti ichi ndichani?") Can name at least five objects in the box of items eg. Bicycle, spoon, cup , ball, pencil, car, bottle, chitenje, plate, beans. Say to the child "What is this?" Give the child all 10 items and see how many he or she can do. Answer YES if the child can do at least FIVE (5) items. Doesn't matter which ones.

20. **Knows his or her first name -** can say it: (Mwana amadziwa dzina lake (amatha kutchula dzina lake) Ask the child for their name. Passes if can **say** their first name.

21. **Knows actions of objects e.g. "which one do you use for sweeping?" Which one is for drinking?** (Amazindikira ntchito ya zinthu monga……. "Chodyera phala ndi chiti?". "Chosesera ndi chiti?" "Chomwera madzi ndi chiti?" Put out a few different objects from the basket and ask questions above or similar ones and see if the child can point to the right objects.

22. **Identifies objects (can give you objects you have named) in the basket – at least 10.** Bicycle, spoon, cup, ball, car, bottle, chitenje, little broom, plate, pencil.) Amazindikira zinthu monga... njinga, sipuni, kapu, mpira, galimoto, mbale. Kumuuza kuti…..) "Ndi patse……………." Say to the child "Give me the spoon, cup....etc" . Count how many out of 10 the child has done. Make sure at least 10 objects are put out. Answer YES if child can identify at least 10 of the ten objects. Doesn't matter which ones.

23. **Names (can say it) 10 objects in the basket: (**Amatchula maina a zinthu zimene zili m'bokosi. Kumfunsa mwana kuti ichi ndichani?") Can name at least five objects in the box of items eg. Bicycle, spoon, cup , ball, pencil, car, bottle, chitenje, plate, beans. Say to the child "What is this?" Give the child all 10 items and see how many he or she can do. Answer YES if the child can do at least TEN (10) items. Doesn't matter which ones.

24. **Is able to categorise things**. For example, ask the child – "Tell me some things that you eat, Tell me some animals that you know…." Kumufunsa mwana…"Ndiuze zinthu zimene umadya…"or "Ndiuze nyama zimene umadziwa…." (Need to be able to do one of these).

25. **Is able to follow a three stage command. For example, "stand up, clap your hands and go over there" or "go over there and collect that cup on the ground and give it to your mum**" Amatha kupanga zinthu zitatu zimene wauzidwa, monga "imilira, womba mmanja ndi pita uko……."

26. **Is able to tell you the use of objects: Knows "What do you do with a spoon, a bicycle, a brush?"** (Funsani Umatani ndi njinga? Umatani ndi pensulo? Pamafunika mayankho monga, timakwera njinga, timajambulira zithunzi, timamwera madzi. Need to have action word included in the answer.

27. **Can do remember back 2 syllables when said to the child :**(Akhonza kukumbukira zilembo, chilembo, chimodzi, ziwiri, zitatu, zinayi) Say to the child. "When I say this, copy me….Pa, Chi, Tu, Go". Say to the child, say "pa", then say to the child "say pa, chi", then say to the child "say pa, chi, tu", then say to the child "say pa, chi, tu, go". See how

far the child can get eg. How many they can do. Answer YES to this if they can at least do the first TWO eg. Pa and Pa, Chi... They get a YES here if they can do more.

28. **Knows 2 of 3 questions relating to the understanding certain concepts e.g. What do you do when you are hungry? What do you do when you are tired? What do you do when you are cold?** (Funsani …umatani ukamva njala? Umatani ukatopa? Umatani ukzizira?)

29. **Understands the adjectives such as "faster" by answering "Which goes faster, a car or a bicycle**?" Amadziwa yankno la funso monga "kodi galimoto ndi njinga chimathamanga kwambiri ndi chiani?

30. **Can do remember back 4 syllables when said to the child :**(Akhonza kukumbukira zilembo, chilembo, chimodzi, ziwiri, zitatu, zinayi) Say to the child. "When I say this, copy me….Pa, Chi, Tu, Go". Say to the child, say "pa", then say to the child "say pa, chi", then say to the child "say pa, chi, tu", then say to the child "say pa, chi, tu, go". See how far the child can get eg. How many they can do. Answer YES to this if they can do all FOUR (4) eg. up to Pa, Chi, Tu, Go..

31. **Can understands prepositions and follow tasks related to this e.g. Put the bean under the cup, put it on the cup, put it next to the cup, put it behind the cup.** (Mufunse mwana aike –pansi, pamwamba, kuseli, pakati. Angathe kuchita zinthu zitatu mwa zinthu zimene zili pamwambapa. Needs to be able to do at least 3 of the 4 of these.

32. **Understands the concept of opposites e.g.** A boy is big, a baby is……, If the sun comes up in the day, the moon comes up ….. (Amadziwa mawu otsutsana a zinthu monga, abambo ndi aankulu, mwana ndi… .. "Ngati dzuwa limatuluka masana, mwezi, umatuluka ……." (must do 2 of these)

33. **Knows quantities – up to 3 –** "how many are these?" (Amadziwa kuchuluka kwa zinthu zokwana zisanu. Mwachitsanzo ukafunsa kuti izi ndi zingati?) Put bricks on the table and ask the child. Can you count these? How many are there? Write down the maximum the child could do. Answer YES to this if they can count 3 or more.

34. **Knows quantities – up to 5 –** "how many are these?" (Amadziwa kuchuluka kwa zinthu zokwana zisanu. Mwachitsanzo ukafunsa kuti izi ndi zingati?) Put bricks on the table and ask the child. Can you count these? How many are there? Write down the maximum the child could do. Answer YES to this if they can count 5 or more.

SOCIAL:

1. **Smiles, but not at a particular person** (Amamwetulira payekha popanda munthu kapena popanda kanthu.). Smiles, but not always sure whether it is in response to someone else or not. Can ask “How does your baby smile?”

2. **Smiles in response to a person:** (Amamwetulira anthu) With the child in mum’s arms or on his or her back, see if the baby will smile in response to the mum’s or your smile and vocalisations.

3. **Frolics with mother or caregiver (moves body in response to another person):** (Amagwedeza thupi kusonyeza kukondwa ndi anthu ena) See if the child moves his or her body in response to being played with by mum or guardian or person who is playing with child.

4. **Frolics alone, plays around moving body, kicking legs in a happy way:** (Amasewera yekha, kugwedeza thupi kuponyaponya miyendo mosangalala) Lies on his or her back and plays around with self moving body and kicking legs happily. Can ask “Kodi mwana amasewera motani?”

5. **Recognises or calms and quiets with caregivers/known family members:** (Amazindikira abale ndikukhala omasuka ndi omwe amamusamalira komanso abale ake. Funsani..”Kodi mwana amazindikira mayi ndi abale akamalira amasiya akawaona?”). Stops crying or quiets when mum or another known family member takes the baby.

6. **Will take phala from a spoon when fed by a caregiver:** (Amadya phala kuchokera pa supuni yomwe womusamalira wayigwira) Is able to eat porridge off a spoon when given it by mum/caregiver, but can not yet hold a spoon. Can eat it off mother’s fingers rather than spoon if this is what is usually used by mother.

7. **Helps to hold a cup while mum gives a drink:** (Naye amagwira kapu akamamumwetsa madzi). Can not yet manage to drink from a cup by self, but will put hand up to cup when mum puts the cup to his or her lips.

8. **Puts arms up or indicates in some way that they want to be picked up:** (Amadziwongola kuti anyamulidwe.) Baby deliberately shows that they want to be picked up by either stretching out arms or trying to lift self towards her.

9. **Can hold a spoon with phala but not get to mouth well:** (Amagwira supuni ya phala koma osayibweretsa kukamwa kuti adzidyetse) Can hold a spoon in hands when being fed and may try to put the spoon with phala in mouth, but can not do it very well. Need to be clear that it is a spoon with food on it.

10. **Drinks form a cup well without spilling:** (Amamwera mkapu opanda kudzitayira) Is able to pick up a cup of water half full and drink from it without spilling any.

11. **Is able to indicate, by pointing, that they want something:** (Amatha kuloza chinthu chimene akufuna) Can show by either pointing or maybe by simple language/noises that they want a particular thing eg. Pointing to the water/cup when they want a drink.

12. **Can the child eat by picking nsima from a plate in morsels that mum has made:** (Amakhoza kutola nsima yekha yomwe amayi ake amutemera. "Kodi mwana amatha kudya pagulu potola mbamu za nsima zomwe amayi ake amutemera kale?") Picks up the nsima in little small portions that mum has separated from the main bit of nsima. Can hold them in his or her hand and put to mouth. Recognises it is food, not just grasping it when put in hand. "Kodi mwana amatha kudya pagulu potola mbamu za nsima zomwe amayi ake amutemera kale?"

13. **Puts hands out to have them washed by mum:** (Amapereka manja kwa mayi kuti asambitsidwe, pakudya) Can not wash hands by self, but understands that he or she needs to have them washed before eating, therefore helps by putting or holding hands out when mum washes them. Not just having them washed in any way, but must understand that they are having them washed.

14. **Can hold a spoon and take phala by self, but spills some:** (Amatenga supuni ndikudzidyetsa phala, kungozitayira pang'ono) Able to feed self with a spoon, but not that well. Spoon sometimes even turns over and spills some, but child enjoys feeding self.

15. **Indicates in some way that they need to go for a poo/pee, for example by crying, pulling at pants or saying something:** (Amaonetsa zizindikiro zoti akufuna kukabiba kapena kukodza monga kulira, kukoka kabudula kapena kunena mawu ena ake) As explained in instructions.

16. **Wants to join in with singing games:** (Amafuna kulowa nawo masewero oyimbayimba ndi anzawo) Is not yet able to do the singing games, but likes them and wants to be part of it even in a small way. "Kodi amafuna kusewera masewero a nyimbo omwe ana okulirapo akusewera ngakhale iye sangathe?"

17. **Able to greet either by extending hand or verbally:** (Amatha kupereka moni wapamanja kapena wamau) Has learned to put out hand to greet someone or can say "moni" or similar greeting.

18. **Sharing things, including food with others:** (Amagawana zinthu ndi anzawo kuphatikiza zakudya) Understands to share things with others, for example if with friends

or other family members, will share food that he or she is given. “Kodi mukampatsa mwana wanu nthochi kapena chakudya chozuna, amagawira anzake?”

19. **Does a poo or pees by themselves without wetting their pants:** (Amatha kubiba kapena kukodza payekha osaipitsa zovala) Is able to know that they need to pee or pass a stool and do it without wetting or soiling themselves. Never wets self.

20. **Can feed self phala off a spoon well without spilling:** (Amadya phala ndi supuni osadzitayira) Can use a spoon well and does not spill or make a mess when using a spoon.

21. **Can make own morsels of nsima and put in mouth, often with soft relish:** (Amakhonza kutema mbamu za nsima ndi kuika mkamwa (makamaka ndi ndiwo zofewa) Can separate balls of nsima from main portion and form into a ball to put into mouth and dip it into the relish. Does not need to be making a perfect ball.

22. **Able to undress by themselves (take off even just 1 item of clothing, such as shorts):** (Amatha kuvula zovala payekha monga kabudula, dilesi kapena siketi) Can take off any item of clothing by themselves. Need to be able to remove it completely. Can be just one item.

23. **Wants to go and visit a friend’s house (shows independence)**: (Amafuna kupita kukacheza kwa anzawo paokha)

24. **Can go to the toilet by themselves anywhere: (**Amachita chimbudzi payekha) Can go and do a poo or a pee by themselves without help, but anywhere eg. Outside, not necessarily on the toilet.

25. **Can eat food/relish with bits in it or bones:** (Amakhonza kudya chomwe chili ndi zinyenyeswa kapena mafupa) Is able to eat for example kapenta fish with bones in it or maybe tangerines with seeds.

26. **Is able to dress but not completely:** (Amatha kuvala payekha chovala ngakhale osakwanitsa bwino lomwe) Is able to put on at least one article of clothing, for example a T-shirt or skirt.

27. **Washes hands well by self before/after eating:** (Amasamba mmanja yekha asanadye chakudya komanso akamaliza kudya) Is able to wash his or her hands without any help before and after eating.

28. **Knows to keep quiet at important meetings or ceremonies**: (Amadziwa kuti ayenera kukhala chete pa gulu la anthu akulu eg. Kutchalichi, pamaliro, pamsonkhano.

29. **Does household chores or helps father or mother in a useful way**: eg. Drawing water/hoeing. (Amathandiza ntchito za pakhomo monga kutunga madzi). This can even be just a small amount of help, but not just pretend. Make sure there are tasks that you ask about that boys could do.

30. **Able to dress by themselves completely:** (Amatha kuvala payekha) Can put on clothes without help, may have help only if tying shoelaces, buttoning or zipping things which are hard to do.

31. **Understands the concept of discipline e.g.**. Causes and consequences eg. Knowing that bad words may lead to punishment. Amadziwa zotsatira za kuchita zoipa mwachitsanzo ngati ayankhula mau oipa monga kutukwana.

32. **Plays games with turn taking eg**. Chipapapa/fly (Amasewera ma sewera opatsana mpata monga fulaye, mira, jingo…..“Ngati akusewera masewero opatsana mpata. Kodi amatha kudikira kuti nthawi yake ifike / ikwane?”

33. **Knows how to be respectful to elders:** (Amadziwa kupereka ulemu kwa akuluakulu monga kugwada popereka moni olo kuwodira. Is polite and shows respect when around elders. For example, putting hands together as a sign of respect or kneeling.

34. **Is able to go to the toilet/pit latrine by self:** (Amakhonza kupita kuchimbudzi payekha) Is able to go and use the pit latrine by themselves and does not need any help in any way.
